# Risk factors for pediatric intoxications in the prehospital setting. A geospatial survey

**DOI:** 10.3389/fpubh.2024.1296250

**Published:** 2024-01-25

**Authors:** Calvin Lukas Kienbacher, Guixing Wei, Jason M. Rhodes, Harald Herkner, Dominik Roth, Kenneth A. Williams

**Affiliations:** ^1^Division of Emergency Medical Services, Department of Emergency Medicine, Brown University, Providence, RI, United States; ^2^Department of Emergency Medicine, Medical University of Vienna, Vienna, Austria; ^3^Spatial Structures in the Social Sciences (S4), Population Studies and Training Center (PSTC), Brown University, Providence, RI, United States; ^4^Rhode Island Department of Health, Center for Emergency Medical Services, Providence, RI, United States

**Keywords:** emergency medical services, intoxication, pediatric emergency medicine, drug overdose, alcohol drinking, public health

## Abstract

**Background:**

Socioeconomic factors and the COVID-19 pandemic influence children’s physical and mental health. We aimed to investigate the association between a census tract’s median household income [MHI in United States Dollars ($)] and pediatric intoxications in Rhode Island, the smallest state in the United States of America. Geographical hotspots, as well as interactions with the COVID-19 pandemic, should be identified.

**Methods:**

This study is a retrospective analysis of ambulance calls for pediatric (<18 years) intoxication in Rhode Island between March 1st, 2018, and February 28th, 2022. March 1st, 2020 was considered the beginning of the COVID-19 pandemic. Prehospital data were joined with information from the United States Census Bureau. The census tracts’ case counts and MHI were examined using Poisson regression. Geographical clusters were identified with the Global Moran’s I and local indicators of spatial association tests in ArcGIS Pro (Esri Corporation, Redlands, CA).

**Results:**

Inclusion criteria were met by 208 incidents (48% female, median age 16 (IQR 15 to 17) years). The regression model showed a 0.6% increase (IRR 1.006, 95% CI [1.002, 1.01], *p* = 0.003) in pediatric intoxications for every $ 1,000 increase in MHI. Interaction analysis showed that the effect of MHI was less pronounced during the pandemic (IRR 0.98, 95% CI [0.964, 0.997], *p* = 0.02). Thirty-four (14%) of the 244 census tracts contributed to geographical clusters, which changed after the onset of the pandemic.

**Conclusion:**

Higher median household income could be a risk factor for pediatric intoxications. Geographical hotspots changed with the pandemic.

## Introduction

Socioeconomic status highly influences child health (1). Minors living in poverty have a high risk of dying before adulthood (1). The COVID-19 pandemic has further worsened these circumstances (2).

Self-harm is a problem of increasing global importance in the pediatric population (3–6). Mental health crises could lead to self-treatment or intoxicating oneself with drugs or medications. Current literature suggests that deliberate ingestion of all kinds of psychoactive substances has increased since the beginning of the ongoing COVID-19 pandemic (7).

This is possibly attributed to the fact that COVID-19 is an additional stress factor, especially for already vulnerable populations with limited coping resources (8). Teenagers were found to be an age group particularly at risk (7).

Prior data indicate that the effects of the COVID-19 pandemic on emergency care facilities greatly varied globally and regionally. This also affects the pediatric population, which the American Academy of Pediatrics (AAP) defines as individuals below 18 years of age (9–11). The pandemic has increased emergency medical service (EMS) response times, potentially compounding the risk of poor outcomes for pediatric patients with intoxications (9). In other places, EMS calls and hospital admissions rapidly declined (9, 12). The population may have been more reluctant to call 9-1-1 or seek care at an emergency department as COVID-19 surged (13). Both circumstances can lead to patients presenting later in their course of illness and, therefore, in a worse clinical condition: Individuals suffering from a medical emergency without receiving adequate first aid immediately are likely to deteriorate swiftly. This might be reflected by altered vital signs, Glasgow Coma Scale (GCS) score, and the clinical gestalt at EMS arrival (14). A complex model investigating patient (i.e., vital signs, clinical impression of prehospital professionals) and operational, EMS-centered (i.e., time intervals) risk factors might therefore deliver valuable clues about the timeliness of care of an ambulance system.

Given the importance of demographic and socioeconomic risk factors and the stressing effect of the ongoing COVID-19 pandemic, it is helpful for ambulance providers and policymakers to anticipate the characteristics of their population at risk to properly allocate their resources. These properties comprise clinical characteristics, geographical clusters of disease entities, and their eventual dynamics. Targeted measures include redirecting EMS resources and establishing first-aid training programs for potential lay bystanders.

Rhode Island is the smallest of the United States of America (US). It has approximately one million inhabitants, and its population demography is around the national average (15). Rhode Island is similar to many cities worldwide in terms of geographic and demographic characteristics. These sociodemographic characteristics are published by the US Census Bureau based on census tracts, i.e., small districts with similar sizes of population (16). The state’s EMS services upload their patient records to a central database overseen by the Rhode Island Department of Health. All reports are stored following the principles of the National EMS Information System (NEMSIS) (17, 18). Rhode Island EMS patient population data can be accessed using filters for categorical (e.g., coded diagnoses) and non-categorical (e.g., trigger words in free text narratives) information. The first cases of COVID-19 in Rhode Island were diagnosed on March 1st, 2020, which is considered as the beginning of the pandemic in the state (19). There was an immediate impact on public health.

Methods of data analysis include non-spatial and geospatial statistics. Common measures of the latter are the Global Moran’s I and local indicators of spatial association (LISA) tests. Both examine the distribution pattern of data points in a geographical area. However, Moran’s I examines the entire map of interest, while LISA investigates individual neighborhoods (20, 21). Clusters, i.e., neighbors with similar characteristics, and outliers, i.e., areas with different properties, can thus be identified. High-high and low-low clusters are areas with similarly high or low rates of an event, respectively. High-low outliers are regions with high rates surrounded by neighbors with low rates. Conversely, low-high outliers are areas with low rates next to regions with high rates (20).

The primary aim was to investigate possible associations between the economic risk factors of median household income (MHI) and child poverty rate, with rates of prehospitally encountered pediatric intoxications. Our secondary aim was to identify high-and low-risk geographical areas. Third, the influence of the COVID-19 pandemic, with its onset on March 1st, 2020, on the condition and clinical presentation of patients was examined. Fourth, the association of the pandemic with both patient-and EMS-centered outcomes was investigated.

## Materials and methods

This study is a retrospective analysis of Rhode Island’s EMS calls for acute intoxications in individuals below the age of 18 within the state of Rhode Island between March 1st, 2018, and February 28th, 2022. We distinguished between the periods before and after the beginning of the COVID-19 pandemic on March 1st, 2020.

We filtered for alcohol, heroin, methamphetamine, opioid intoxications, and substance overdose in general in NEMSIS using a proprietary search engine [biospatial tool (22, 23)]. Afterward, all patient care narratives were screened manually to ensure that inclusion criteria were met. Inconclusive calls, non-primary, non-9-1-1 responses, and victims of mass casualty incidents were excluded. Data acquisition and screening were performed by a board-certified EMS physician, who is also a licensed paramedic. Based on the geolocations of the EMS calls, the dataset was joined with the most recent year 2020 census tract geographical, demographical, and socioeconomic data in ArcGIS Pro 2.9.3 (Esri Corporation, Redlands, CA), which was used for spatial analysis. This includes the population under 18 years of age, the percentage of children living in poverty, and MHI, measured in 2020 inflation-adjusted United States Dollars ($). This information was provided by the US Census Bureau (24, 25). For ease of analysis purposes, MHI was converted to units of $1,000.

Poisson regression models with counts as dependent and the economic risk factors as independent variables were used with a census tract as a unit of analysis. Interaction analyzes were undertaken for the pandemic with MHI and child poverty rate. Individual random-effects linear regression models were used to investigate the association between EMS intervals as the dependent variables and the COVID-19 pandemic, as well as the socioeconomic risk factors as independent variables with the cases as units of analysis. Results are presented as point estimates, 95% confidence intervals (CI), and *p*-values.

Global Moran’s I statistics and LISA for EMS call rates within census tracts (number of calls per 10,000 people under 18 years of age) were computed for the entire observation period and the subperiods before and after the beginning of the COVID-19 pandemic. The Queen contiguity spatial relationship, defining neighbors as census tracts sharing common edges or borders, was used to construct the row-standardized weight matrix across all spatial analyzes. Local clusters and outliers were identified.

Four census tracts without any population at risk (identifiers 44,003,980,000, 44,005,990,000, 44,009,990,100, and 44,009,990,200) were excluded from the study. Two census tracts being islands (identifiers 44,009,041,500 and 44,005,041,300) do not have neighbors and were excluded from the geospatial analysis.

Differences in initial vital signs frequently measured by EMS in children during the primary survey [i.e., heart rate, respiratory rate, body temperature, blood sugar levels, and peripherally measured oxygen saturation (SpO_2_)] between, before, and after the onset of the COVID-19 pandemic were investigated. Normal parameters were defined as follows:

- Heart rate and respiratory rate: ≥10th and ≤ 90th percentiles, according to the commonly referenced work of Fleming et al. (26), in order to achieve comparability across different age groups.
- Body temperature ≥ 36.0 and ≤ 37.9°C.
- Blood sugar levels ≥70 and ≤ 150 mg/dL.
- SpO_2_ ≥ 95%.

Patients deemed “critical” or “emergent” by EMS crews were defined as unstable, and those assessed to be of “lower acuity” as stable. Proportions of the study population with normal and abnormal vital signs, and initial stability (stable vs. unstable) as assessed by the ambulance crews were compared between, before, and after the beginning of the pandemic using the chi-squared test.

Differences in EMS-related intervals were examined using median regression. They were defined as follows:

- Response interval: From the EMS unit being notified to arrival at the patient.
- On-scene interval: From arrival at the patient to leaving the scene.
- Transport interval: From leaving the scene to arriving at the destination.
- Back-to-service interval: From arriving at the destination to reporting the unit being back in service. This interval comprises patient handover, decontamination, doffing of personal protection equipment, and cleaning the vehicle.
- Overall mission interval: Sum of all intervals.

The response, transport, and back-to-service times were log-transformed to better fit the regression model. All test results with a two-sided *p*-value of ≤0.05 were considered to be statistically significant. MS Excel 16.62 (Microsoft Corporation) was used for data curation. Stata 17MP (Stata Corporation) was utilized for non-spatial analyzes. All statistical analyzes were conducted involving a geospatial analyst and a clinical epidemiologist. The RECORD statement for our manuscript can be found in Supplementary Table S1 (27).

The Rhode Island Department of Health’s institutional review board approved the study protocol with an exemption from full review (vote #2022-01). This project was conducted in accordance with the principles of the Declaration of Helsinki.

## Results

Inclusion criteria were met by 208 emergency calls (99 (48%) female). The patients’ median age was 16 (IQR 15 to 17) years (see Table 1 for baseline characteristics of the study population). Four census tracts had no population at risk and were excluded. The statewide rate of pediatric intoxications was 6 before and 5 per 10,000 children after the beginning of the pandemic, respectively. The median rates per census tract were 0 (IQR 0 to 8) before and 0 (IQR 0 to 9) per 10,000 children since the onset of COVID-19.

**Table 1:** Characteristics of the study population.

	Overall (*N* = 208)	Before COVID-19 (*n* = 115)	Since COVID-19 (*n* = 93)
Female, *n* (%)	99 (48)	47 (41)	52 (56)
Age, years, median (IQR)	16 (15 to 17)	16 (16 to 17)	16 (15 to 17)
Age groups, *n* (%)			
Teenager (13–17 years)	201 (97)	111 (97)	90 (97)
Other age groups (1–12 years)	6 (3)	4 (3)	2 (2)
Suspected agent, *n* (%)			
Alcohol	174 (84)	97 (84)	77 (83)
Cannabis	26 (13)	19 (17)	7 (8)
Medications	9 (4)	0 (0)	9 (10)
Opioids	5 (2)	3 (3)	2 (2)
Other	5 (2)	4 (3)	1 (1)
unknown	17 (8)	10 (9)	7 (8)
Multiple substances	28 (13)	18 (16)	10 (11)

Percentages rounded to integers. IQR interquartile range, *N*/*n* number of subjects.

**Figure 1:**
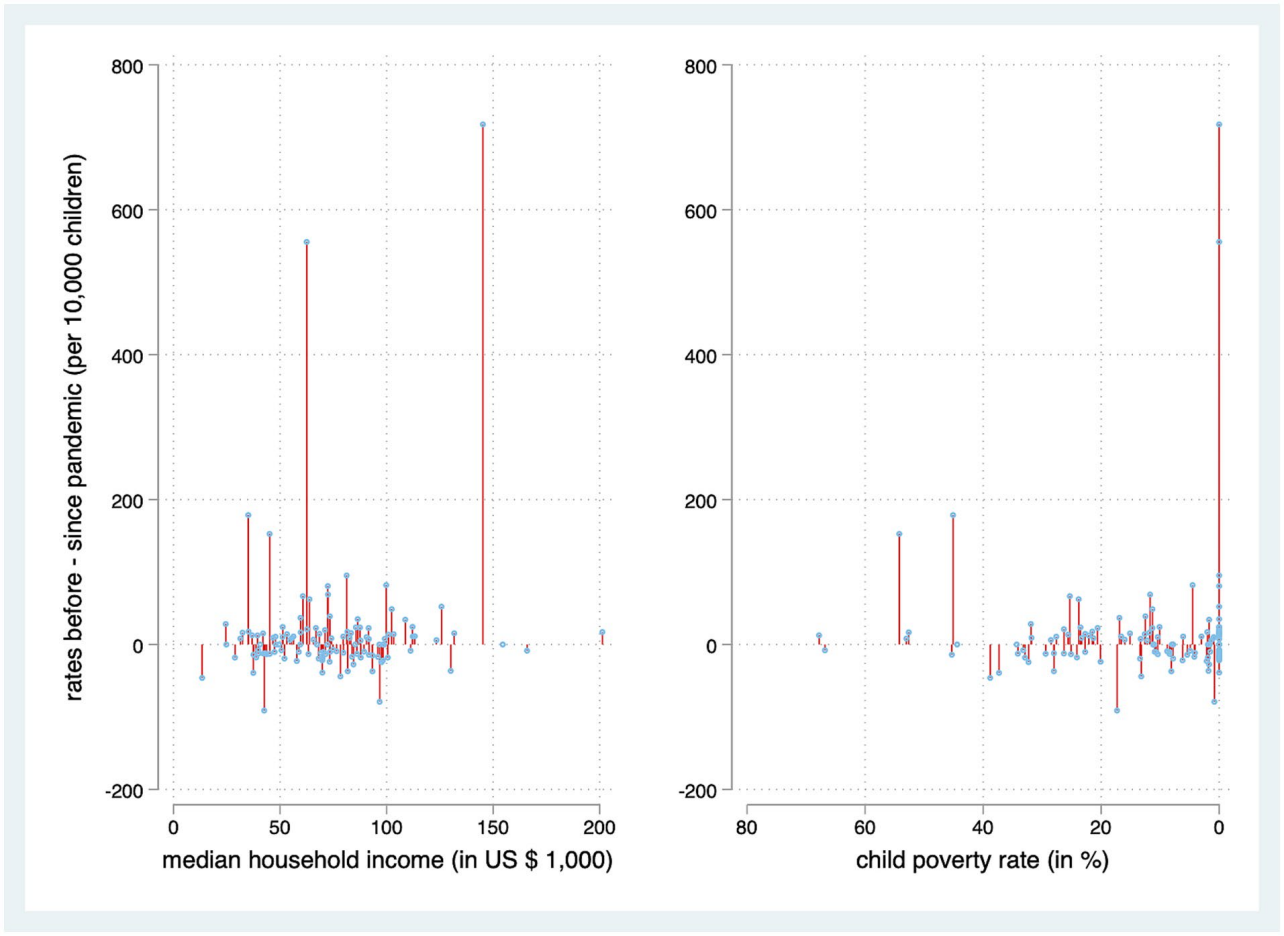
Association between economic risk factors associated with census tracts and their rates of pediatric intoxications. Census tracts without cases are omitted for better visualization.

The Poisson regression model on a census tract basis revealed an incidence rate ratio (IRR) of 1.006 (95% CI [1.002, 1.01]; *p* = 0.003) for pediatric intoxications for each $1,000 increase in MHI. Thus, higher MHI was associated with higher rates of the condition. Higher child poverty percentages were insignificantly associated with lower rates of pediatric intoxication (IRR 0.996, 95% CI [0.987, 1.005]; *p* = 0.35). Interaction analysis showed that the effect of MHI was less pronounced during the pandemic (IRR 0.98, 95% CI [0.964, 0.997], *p* = 0.02)/ This effect was not present regarding the child poverty rate (IRR 0.969, 95% [0.919, 1.022], *p* = 0.24). Figure 1 displays the relationship between economic risk factors and call rates.

Excluding two islands, 244 census tracts with 207 incidents underwent geospatial statistical analysis. Global Moran’s I testing revealed that cases of pediatric intoxication occurred in geographical clusters over the entire observation period (z-score 6.5; *p* < 0.001), as well as in the subperiods before (z-score 5.4; *p* < 0.001) and after (z-score 2.9; *p* < 0.001) the beginning of the COVID-19 pandemic. Clustering was less pronounced after the pandemic than before. The high-risk regions for pediatric intoxications shifted over time. The greater, less wealthy area of the capital, Providence, in the northeast, was a diverse and fluctuating region in terms of intoxication rates. The likewise less wealthy mid-west of the state faced fewer cases than the east.

**Figure 2:**
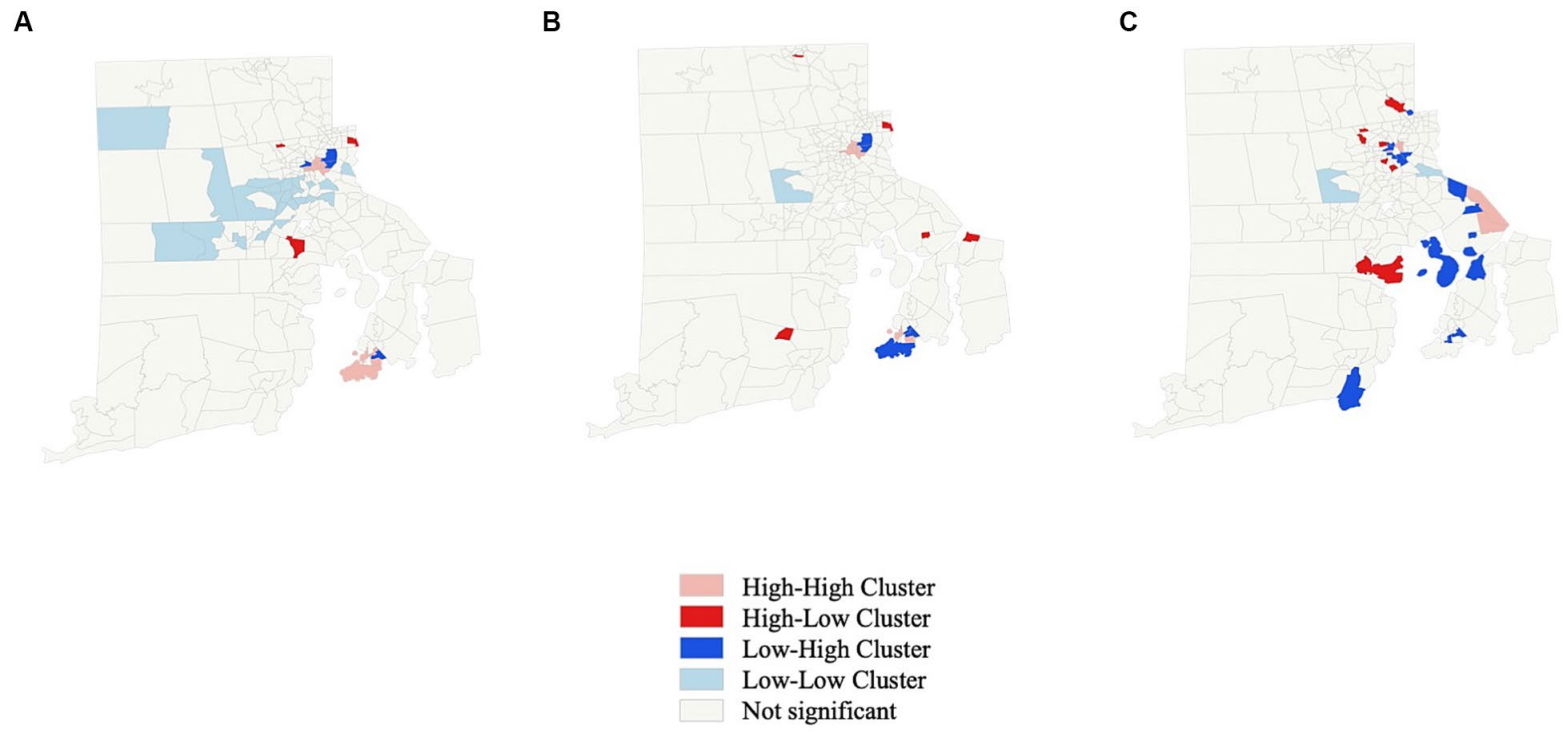
Local indicators of spatial association (LISA) for rate of pediatric intoxication on a census tract basis. **(A)** Entire observation period. **(B)** Before beginning of COVID-19 pandemic. **(C)** After beginning of COVID-19 pandemic.

Using LISA to investigate immediate neighborhoods, 23 census tracts encountered low rates of pediatric intoxications and were surrounded by other census tracts with low rates. Eleven census tracts faced high rates and bordered areas with similarly high rates of the condition. In contrast, 3 were census tracts with higher rates of pediatric intoxications surrounded by census tracts with lower rates. Four were identified as census tracts with lower rates, surrounded by areas with higher rates over the entire observation period (see Figure 2A). Figures 2B,C display the LISA results of the two split periods: The geographical pattern on a neighborhood basis also changed between before and after the onset of the pandemic. Again, the greater area of the city of Providence faced shifts of neighborhood hotspots between before and after the beginning of the pandemic.

More patients encountered during the pandemic had an abnormal heart rate (43% vs. 56%; *p* = 0.04). No significant differences regarding any other vital parameters, the proportion of patients with at least one abnormal vital parameter, unstable conditions, or a GCS of less than 15 points were found (see Table 2).

**Table 2:** Findings in primary survey.

	Overall (*N* = 208)	Before COVID-19 (*n* = 115)	Since COVID-19 (*n* = 93)
Emergent or critical, *n* (%)	51 (25)	30 (26)	21 (23)
Any abnormal vital sign, *n* (%)	140 (67)	76 (66)	64 (69)
GCS score below 15 (points), *n* (%)	73 (35)	44 (38)	29 (31)
abnormal vital signs, *n* (%)			
Heart rate	102 (49)	50 (43)	52 (56)
Respiratory rate	65 (31)	37 (32)	28 (30)
SpO_2_	3 (1)	1 (1)	2 (2)
Blood glucose	23 (11)	15 (13)	8 (9)
Body temperature	1 (0)	0 (0)	1 (0)

Percentages rounded to integers. GCS Glasgow Coma Scale, *N*/*n* number of subjects, SpO_2_ peripheral oxygen saturation.

Response times [6 (IQR 5 to 9) vs. 8 (5 to 10) minutes; *p* = 0.03] and on-scene times [11 (IQR 7 to 15) vs. 14 (9 to 20) minutes; *p* = 0.02] were significantly shorter after the onset of the pandemic. Transport, back-to-service, and overall mission times remained unaltered. Please see Table 2 for details.

Higher MHI was associated with longer response (coefficient 0.004, 95% CI [0.001, 0.006]; *p* = 0.004), overall mission (coefficient 0.15, 95% CI [0.02, 0.27]; *p* = 0.02), and transport times (coefficient 0.01, 95% CI [0.01, 0.01]; *p* < 0.001). The pandemic (coefficient-3.6, 95% CI [−6.1, −1.2]; p = 0.004) was associated with shorter times spent on scene, and higher MHI (coefficient 0.06, 95% CI [0.02, 0.09]; *p* = 0.001) was associated with longer times spent on scene. Over the entire study period, patients in high-high clusters experienced longer on-scene (coefficient 5.5, 95% CI [2.2, 8.9]; *p* = 0.001) and shorter transport times (coefficient-0.3, 95% CI [−0.6, −0.1]; *p* = 0.02). The former was still the case after adjusting for the individual LISA characteristics before and after the pandemic, respectively (coefficient 6.6, 95% CI [3, 10.1]; *p* < 0.001). The complete Poisson and linear regression models can be found in Supplementary Table S2.

Alcohol (84%) was the most often suspected substance causing the intoxication, followed by cannabis (13%) and various medications (4%). All nine incidents involving the latter took place after the beginning of the pandemic. Although suspected opioid overdose was rare in this study’s population (*n* = 5, 2%), nine (4%) patients were treated with naloxone prehospitally. This number includes 3 (60%) of the children with suspected opioid overdose. Twenty-eight individuals (13%) ingested multiple substances (see Table 3).

**Table 3:** Comparison of emergency medical service-related times before and after the beginning of the COVID-19 pandemic.

	Overall	Before COVID-19 pandemic	After beginning of COVID-19 pandemic	before vs. after beginning of COVID-19 pandemic, median [95% CI]
Response time, minutes, median (IQR), *n* = 208	7 (5 to 10)	8 (5 to 10)	6 (5 to 9)	1 [0, 3]^a^
Time on-scene, minutes, median (IQR), *n* = 189	12 (8 to 18)	14 (9 to 20)	11 (7 to 15)	3 [1, 6]^b^
Transport time, minutes, median (IQR), *n* = 181	9 (5 to 15)	9 (5 to 14)	11 (6 to 17)	−2 [−5, 1]
Back-to-service time, minutes, median (IQR), *n* = 181	21 (14 to 35)	21 (14 to 35)	22 (14 to 33)	−1 [−6, 4]
Overall mission time, minutes, median (IQR), *n* = 208	52 (40 to 66)	53 (41 to 69)	51 (39 to 65)	3 [−4, 9]

All values rounded to integers. ^a^*p* = 0.03, ^b^*p* = 0.02. IQR interquartile range, n number of subjects, 95% CI 95% confidence interval.

## Discussion

The findings indicate that census tracts with a higher MHI have higher rates of pediatric intoxications. Events are geographically clustered, with a weaker pattern after than before the pandemic. Hotspots are not stable geographical areas but are subject to change over time.

Our results deviate from those of Salmi et al., who found that children living in less wealthy circumstances were, in general, more likely to be encountered by EMS (28). However, their study did not exclusively focus on intoxications. Chalfin and colleagues showed that higher parental education, which often correlates with income, resulted in less underage binge drinking but had no influence on cannabis consumption (29). Regarding the clustering around urban areas, our results are consistent with the work of Stopka et al. from Massachusetts (30). Additionally, Bearnot et al. (31) found hotspots of drug use in areas facing socioeconomic hardships and around hospitals. However, both studies focused on opioid overdose in the general population.

Fewer records were identified for the period after than before the onset of the pandemic. These findings are in accordance with prior data (9, 10, 12, 13). In contradiction, Raffee and colleagues found higher numbers of poison center activations after the onset of the pandemic in Jordan. This fact raises the question of whether the number of cases in the present study’s population truly decreased or if incidents were just not reported as often as beforehand (32).

Alcohol and cannabis, sometimes taken in combination, were the most common suspected agents in this present study’s mainly adolescent population. The mechanisms of ingestion of these two substances, orally or by smoking, are highly suggestive of a predominantly intentional consumption. Both decreased after the onset of the pandemic. This could be related to the fact that these substances were then more difficult to obtain. In contrast, all intoxications involving medication occurred after the onset of the pandemic. The unavailability of alcohol and cannabis may have led to self-treatment using pharmaceuticals. This theory is supported by the findings of Wallis et al., indicating that cannabis is frequently used to self-medicate to relieve anxiety and pain in young adults (33). Medicinal products might be viewed as an alternative when cannabis is less easily accessible.

Interestingly, almost twice as many patients as those in whom opioid intoxication was suspected received naloxone. This fact is likely related to successful multilingual overdose awareness campaigns, which have been launched in the light of rising numbers of fatal cases (34, 35). When in doubt, the antidote might rather be administered than withheld.

Our data show that response times were longer in areas with higher MHI. This is likely related to the lower population density of richer suburban areas. Our results were similar to those of Eskol et al., who also found longer times spent on scene since the beginning of the COVID-19 pandemic, with all other prehospital intervals being unaltered (36). This issue may be related to the additional time needed to put on PPE during the pandemic, which was required by the Rhode Island Department of Health. Considering the shorter EMS-related intervals but a largely unaltered proportion of patients with abnormal initial vital signs, patients were likely primarily not suffering from worse clinical conditions before compared to after the onset of the pandemic. In this context, the clinical relevance of the statistically significant differences in EMS intervals between the subperiods remains debatable.

This study has two important limitations. First, the generalizability of its findings for regions outside the US might be threatened by legal issues: Alcohol was the most often consumed substance in this study’s population. Laws regulating the legal drinking age vary greatly across countries. Being afraid of liability implications, children suffering from alcohol intoxication themselves or their bystanders could be inherently more reluctant to call 9-1-1 in the US than in other countries. We used simplified models to elaborate our study question. Aside from the important risk factors taken into account, there are likely many other circumstances influencing the risk of pediatric intoxication in a region. These may include the presence of child protection services and social work, as well as the individual political environment.

Second, the economic risk factors chosen for the analysis are only available on a census tract basis. The actual financial background of the individual cases remains unknown. However, this approach is well-established and has been frequently used by other researchers in the past (37–40).

## Conclusion

Higher economic status is possibly associated with increased rates of prehospitally encountered pediatric intoxications. The COVID-19 pandemic led to shorter response and on-scene intervals for emergency medical service personnel responding to these incidences.

## Data availability statement

The datasets presented in this article are not readily available because data cannot be shared due to IRB regulations. Requests to access the datasets should be directed to calvin.kienbacher@meduniwien.ac.at.

## Ethics statement

The studies involving humans were approved by the Institutional Review Board of the Rhode Island Department of Health. The studies were conducted in accordance with the local legislation and institutional requirements. The ethics committee/institutional review board waived the requirement of written informed consent for participation from the participants or the participants’ legal guardians/next of kin because the project is a low-risk retrospective chart review.

## Author contributions

CK: Conceptualization, Data curation, Formal analysis, Investigation, Methodology, Project administration, Resources, Software, Supervision, Validation, Visualization, Writing – original draft. GW: Data curation, Formal analysis, Investigation, Methodology, Resources, Software, Validation, Writing – review & editing. JR: Investigation, Methodology, Project administration, Resources, Supervision, Validation, Writing – review & editing. HH: Conceptualization, Formal analysis, Investigation, Methodology, Resources, Software, Validation, Writing – review & editing. DR: Investigation, Methodology, Writing – review & editing. KW: Conceptualization, Investigation, Methodology, Project administration, Supervision, Validation, Writing – review & editing.
