# Supplementary material for: Risk factors for pediatric intoxications in the prehospital setting. A geospatial survey
Source: Front Public Health. 2024 Jan 25;12:1296250. doi: 10.3389/fpubh.2024.1296250 (PMC10851149; doi:10.3389/fpubh.2024.1296250)
Supplement: Supplementary file 2 [file Table_2.docx]

**Supplementary Table 2. Regression models.**

**Poisson regression**

| ambulance calls | Coef. | | St.Err. | t-value | | p-value | [95% Conf | | Interval] | | Sig |
| --- | --- | --- | --- | --- | --- | --- | --- | --- | --- | --- | --- |
| MHI | 1.006 | | .002 | 2.92 | | .003 | 1.002 | | 1.01 | | *** |
| Constant | .001 | | 0 | -40.69 | | 0 | 0 | | .001 | | *** |
|  | | | | | | | | | | | |
| Mean dependent var | | 0.846 | | | SD dependent var | | | 1.493 | |  |  |
| Pseudo r-squared | | 0.010 | | | Number of obs | | | 246 | |  |  |
| Chi-square | | 8.064 | | | Prob > chi2 | | | 0.005 | |  |  |
| Akaike crit. (AIC) | | 822.008 | | | Bayesian crit. (BIC) | | | 829.019 | |  |  |
| **** p<.01, ** p<.05, * p<.1, MHI median household income in US$ 1,000, child poverty rate in %* | | | | | | | | | | | |
|  | | | | | | | | | | | |

**Poisson regression**

| ambulance calls | Coef. | | St.Err. | t-value | | p-value | [95% Conf | | Interval] | | Sig |
| --- | --- | --- | --- | --- | --- | --- | --- | --- | --- | --- | --- |
| child poverty rate | .996 | | .004 | -0.93 | | .354 | .987 | | 1.005 | |  |
| Constant | .001 | | 0 | -72.15 | | 0 | .001 | | .001 | | *** |
|  | | | | | | | | | | | |
| Mean dependent var | | 0.846 | | | SD dependent var | | | 1.493 | |  |  |
| Pseudo r-squared | | 0.001 | | | Number of obs | | | 246 | |  |  |
| Chi-square | | 0.882 | | | Prob > chi2 | | | 0.348 | |  |  |
| Akaike crit. (AIC) | | 829.190 | | | Bayesian crit. (BIC) | | | 836.201 | |  |  |
| **** p<.01, ** p<.05, * p<.1, MHI median household income in US$ 1,000, child poverty rate in %* | | | | | | | | | | | |
|  | | | | | | | | | | | |

**Poisson regression**

| ambulance calls | Coef. | | St.Err. | t-value | | p-value | [95% Conf | | Interval] | | Sig |
| --- | --- | --- | --- | --- | --- | --- | --- | --- | --- | --- | --- |
| MHI | 1.017 | | .008 | 2.07 | | .038 | 1.001 | | 1.033 | | ** |
| child poverty rate | 1.031 | | .024 | 1.27 | | .204 | .984 | | 1.08 | |  |
| MHI#child poverty rate | 1 | | 0 | -0.82 | | .414 | .999 | | 1.001 | |  |
| COVID-19 | 4.805 | | 3.361 | 2.24 | | .025 | 1.22 | | 18.924 | | ** |
| COVID-19#MHI | .98 | | .008 | -2.36 | | .018 | .964 | | .997 | | ** |
| COVID-19#child poverty rate | .969 | | .026 | -1.17 | | .241 | .919 | | 1.022 | |  |
| COVID-19#MHI#child poverty rate | 1 | | .001 | 0.55 | | .585 | .999 | | 1.001 | |  |
| Constant | 0 | | 0 | -14.63 | | 0 | 0 | | 0 | | *** |
|  | | | | | | | | | | | |
| Mean dependent var | | 0.423 | | | SD dependent var | | | 1.005 | |  |  |
| Pseudo r-squared | | 0.025 | | | Number of obs | | | 492 | |  |  |
| Chi-square | | 8.018 | | | Prob > chi2 | | | 0.331 | |  |  |
| Akaike crit. (AIC) | | 1044.580 | | | Bayesian crit. (BIC) | | | 1078.168 | |  |  |
| **** p<.01, ** p<.05, * p<.1, MHI median household income in US$ 1,000, child poverty rate in %* | | | | | | | | | | | |
|  | | | | | | | | | | | |

**Regression results**

| response interval (log) | Coef. | | St.Err. | t-value | | p-value | [95% Conf | | Interval] | | Sig |
| --- | --- | --- | --- | --- | --- | --- | --- | --- | --- | --- | --- |
| MHI | .004 | | .001 | 2.92 | | .004 | .001 | | .006 | | *** |
| Constant | 1.643 | | .105 | 15.61 | | 0 | 1.436 | | 1.849 | | *** |
|  | | | | | | | | | | | |
| Mean dependent var | | 1.927 | | | SD dependent var | | | 0.572 | |  |  |
| Overall r-squared | | 0.048 | | | Number of obs | | | 208 | |  |  |
| Chi-square | | 8.505 | | | Prob > chi2 | | | 0.004 | |  |  |
| R-squared within | | 0.000 | | | R-squared between | | | 0.076 | |  |  |
| **** p<.01, ** p<.05, * p<.1, MHI median household income in US$ 1,000, child poverty rate in %* | | | | | | | | | | | |
|  | | | | | | | | | | | |

**Regression results**

| response interval (log) | Coef. | | St.Err. | t-value | | p-value | [95% Conf | | Interval] | | Sig |
| --- | --- | --- | --- | --- | --- | --- | --- | --- | --- | --- | --- |
| child poverty rate | -.003 | | .003 | -0.94 | | .349 | -.008 | | .003 | |  |
| Constant | 1.962 | | .061 | 32.11 | | 0 | 1.842 | | 2.082 | | *** |
|  | | | | | | | | | | | |
| Mean dependent var | | 1.927 | | | SD dependent var | | | 0.572 | |  |  |
| Overall r-squared | | 0.005 | | | Number of obs | | | 208 | |  |  |
| Chi-square | | 0.878 | | | Prob > chi2 | | | 0.349 | |  |  |
| R-squared within | | 0.000 | | | R-squared between | | | 0.011 | |  |  |
| **** p<.01, ** p<.05, * p<.1, MHI median household income in US$ 1,000, child poverty rate in %* | | | | | | | | | | | |
|  | | | | | | | | | | | |

**Regression results**

| response interval (log) | Coef. | | St.Err. | t-value | | p-value | [95% Conf | | Interval] | | Sig |
| --- | --- | --- | --- | --- | --- | --- | --- | --- | --- | --- | --- |
| COVID-19 | -.051 | | .082 | -0.62 | | .535 | -.211 | | .11 | |  |
| Constant | 1.947 | | .059 | 32.75 | | 0 | 1.831 | | 2.064 | | *** |
|  | | | | | | | | | | | |
| Mean dependent var | | 1.927 | | | SD dependent var | | | 0.572 | |  |  |
| Overall r-squared | | 0.003 | | | Number of obs | | | 208 | |  |  |
| Chi-square | | 0.385 | | | Prob > chi2 | | | 0.535 | |  |  |
| R-squared within | | 0.002 | | | R-squared between | | | 0.001 | |  |  |
| **** p<.01, ** p<.05, * p<.1, MHI median household income in US$ 1,000, child poverty rate in %* | | | | | | | | | | | |
|  | | | | | | | | | | | |

**Regression results**

| on-scene interval | Coef. | | St.Err. | t-value | | p-value | [95% Conf | | Interval] | | Sig |
| --- | --- | --- | --- | --- | --- | --- | --- | --- | --- | --- | --- |
| MHI | .057 | | .017 | 3.46 | | .001 | .025 | | .09 | | *** |
| Constant | 9.49 | | 1.418 | 6.70 | | 0 | 6.712 | | 12.269 | | *** |
|  | | | | | | | | | | | |
| Mean dependent var | | 13.918 | | | SD dependent var | | | 8.625 | |  |  |
| Overall r-squared | | 0.061 | | | Number of obs | | | 188 | |  |  |
| Chi-square | | 11.983 | | | Prob > chi2 | | | 0.001 | |  |  |
| R-squared within | | 0.000 | | | R-squared between | | | 0.014 | |  |  |
| **** p<.01, ** p<.05, * p<.1, MHI median household income in US$ 1,000, child poverty rate in %* | | | | | | | | | | | |
|  | | | | | | | | | | | |

**Regression results**

| on-scene interval | Coef. | | St.Err. | t-value | | p-value | [95% Conf | | Interval] | | Sig |
| --- | --- | --- | --- | --- | --- | --- | --- | --- | --- | --- | --- |
| child poverty rate | -.028 | | .037 | -0.75 | | .455 | -.1 | | .045 | |  |
| Constant | 14.342 | | .848 | 16.92 | | 0 | 12.68 | | 16.003 | | *** |
|  | | | | | | | | | | | |
| Mean dependent var | | 13.918 | | | SD dependent var | | | 8.625 | |  |  |
| Overall r-squared | | 0.003 | | | Number of obs | | | 188 | |  |  |
| Chi-square | | 0.558 | | | Prob > chi2 | | | 0.455 | |  |  |
| R-squared within | | 0.000 | | | R-squared between | | | 0.012 | |  |  |
| **** p<.01, ** p<.05, * p<.1, MHI median household income in US$ 1,000, child poverty rate in %* | | | | | | | | | | | |
|  | | | | | | | | | | | |

**Regression results**

| on-scene interval | Coef. | | St.Err. | t-value | | p-value | [95% Conf | | Interval] | | Sig |
| --- | --- | --- | --- | --- | --- | --- | --- | --- | --- | --- | --- |
| COVID-19 | -3.625 | | 1.238 | -2.93 | | .003 | -6.051 | | -1.199 | | *** |
| Constant | 15.576 | | .837 | 18.60 | | 0 | 13.935 | | 17.217 | | *** |
|  | | | | | | | | | | | |
| Mean dependent var | | 13.918 | | | SD dependent var | | | 8.625 | |  |  |
| Overall r-squared | | 0.044 | | | Number of obs | | | 188 | |  |  |
| Chi-square | | 8.574 | | | Prob > chi2 | | | 0.003 | |  |  |
| R-squared within | | 0.041 | | | R-squared between | | | 0.025 | |  |  |
| **** p<.01, ** p<.05, * p<.1, MHI median household income in US$ 1,000, child poverty rate in %* | | | | | | | | | | | |
|  | | | | | | | | | | | |

**Regression results**

| transport interval (log) | Coef. | | St.Err. | t-value | | p-value | [95% Conf | | Interval] | | Sig |
| --- | --- | --- | --- | --- | --- | --- | --- | --- | --- | --- | --- |
| MHI | .008 | | .002 | 5.24 | | 0 | .005 | | .011 | | *** |
| Constant | 1.581 | | .129 | 12.25 | | 0 | 1.328 | | 1.834 | | *** |
|  | | | | | | | | | | | |
| Mean dependent var | | 2.185 | | | SD dependent var | | | 0.673 | |  |  |
| Overall r-squared | | 0.145 | | | Number of obs | | | 181 | |  |  |
| Chi-square | | 27.437 | | | Prob > chi2 | | | 0.000 | |  |  |
| R-squared within | | 0.000 | | | R-squared between | | | 0.210 | |  |  |
| **** p<.01, ** p<.05, * p<.1, MHI median household income in US$ 1,000, child poverty rate in %* | | | | | | | | | | | |
|  | | | | | | | | | | | |

**Regression results**

| transport interval (log) | Coef. | | St.Err. | t-value | | p-value | [95% Conf | | Interval] | | Sig |
| --- | --- | --- | --- | --- | --- | --- | --- | --- | --- | --- | --- |
| child poverty rate | -.013 | | .003 | -3.73 | | 0 | -.019 | | -.006 | | *** |
| Constant | 2.389 | | .075 | 31.97 | | 0 | 2.242 | | 2.535 | | *** |
|  | | | | | | | | | | | |
| Mean dependent var | | 2.185 | | | SD dependent var | | | 0.673 | |  |  |
| Overall r-squared | | 0.080 | | | Number of obs | | | 181 | |  |  |
| Chi-square | | 13.929 | | | Prob > chi2 | | | 0.000 | |  |  |
| R-squared within | | 0.000 | | | R-squared between | | | 0.117 | |  |  |
| **** p<.01, ** p<.05, * p<.1, MHI median household income in US$ 1,000, child poverty rate in %* | | | | | | | | | | | |
|  | | | | | | | | | | | |

**Regression results**

| transport interval (log) | Coef. | | St.Err. | t-value | | p-value | [95% Conf | | Interval] | | Sig |
| --- | --- | --- | --- | --- | --- | --- | --- | --- | --- | --- | --- |
| COVID-19 | .239 | | .092 | 2.61 | | .009 | .059 | | .419 | | *** |
| Constant | 2.087 | | .073 | 28.43 | | 0 | 1.943 | | 2.231 | | *** |
|  | | | | | | | | | | | |
| Mean dependent var | | 2.185 | | | SD dependent var | | | 0.673 | |  |  |
| Overall r-squared | | 0.019 | | | Number of obs | | | 181 | |  |  |
| Chi-square | | 6.794 | | | Prob > chi2 | | | 0.009 | |  |  |
| R-squared within | | 0.069 | | | R-squared between | | | 0.022 | |  |  |
| **** p<.01, ** p<.05, * p<.1, MHI median household income in US$ 1,000, child poverty rate in %* | | | | | | | | | | | |
|  | | | | | | | | | | | |

**Regression results**

| back-to-service interval (log) | Coef. | | St.Err. | t-value | | p-value | [95% Conf | | Interval] | | Sig |
| --- | --- | --- | --- | --- | --- | --- | --- | --- | --- | --- | --- |
| MHI | -.001 | | .003 | -0.31 | | .756 | -.007 | | .005 | |  |
| Constant | 3.022 | | .265 | 11.38 | | 0 | 2.502 | | 3.542 | | *** |
|  | | | | | | | | | | | |
| Mean dependent var | | 2.985 | | | SD dependent var | | | 0.981 | |  |  |
| Overall r-squared | | 0.000 | | | Number of obs | | | 181 | |  |  |
| Chi-square | | 0.096 | | | Prob > chi2 | | | 0.756 | |  |  |
| R-squared within | | . | | | R-squared between | | | 0.001 | |  |  |
| **** p<.01, ** p<.05, * p<.1, MHI median household income in US$ 1,000, child poverty rate in %* | | | | | | | | | | | |
|  | | | | | | | | | | | |

**Regression results**

| back-to-service interval (log) | Coef. | | St.Err. | t-value | | p-value | [95% Conf | | Interval] | | Sig |
| --- | --- | --- | --- | --- | --- | --- | --- | --- | --- | --- | --- |
| child poverty rate | .002 | | .007 | 0.32 | | .747 | -.011 | | .015 | |  |
| Constant | 2.916 | | .141 | 20.72 | | 0 | 2.64 | | 3.192 | | *** |
|  | | | | | | | | | | | |
| Mean dependent var | | 2.985 | | | SD dependent var | | | 0.981 | |  |  |
| Overall r-squared | | 0.010 | | | Number of obs | | | 181 | |  |  |
| Chi-square | | 0.104 | | | Prob > chi2 | | | 0.747 | |  |  |
| R-squared within | | 0.000 | | | R-squared between | | | 0.000 | |  |  |
| **** p<.01, ** p<.05, * p<.1, MHI median household income in US$ 1,000, child poverty rate in %* | | | | | | | | | | | |
|  | | | | | | | | | | | |

**Regression results**

| back-to-service interval (log) | Coef. | | St.Err. | t-value | | p-value | [95% Conf | | Interval] | | Sig |
| --- | --- | --- | --- | --- | --- | --- | --- | --- | --- | --- | --- |
| COVID-19 | .117 | | .13 | 0.90 | | .368 | -.138 | | .372 | |  |
| Constant | 2.889 | | .122 | 23.67 | | 0 | 2.65 | | 3.128 | | *** |
|  | | | | | | | | | | | |
| Mean dependent var | | 2.985 | | | SD dependent var | | | 0.981 | |  |  |
| Overall r-squared | | 0.000 | | | Number of obs | | | 181 | |  |  |
| Chi-square | | 0.810 | | | Prob > chi2 | | | 0.368 | |  |  |
| R-squared within | | 0.021 | | | R-squared between | | | 0.001 | |  |  |
| **** p<.01, ** p<.05, * p<.1, MHI median household income in US$ 1,000, child poverty rate in %* | | | | | | | | | | | |
|  | | | | | | | | | | | |

**Regression results**

| overall mission interval | Coef. | | St.Err. | t-value | | p-value | [95% Conf | | Interval] | | Sig |
| --- | --- | --- | --- | --- | --- | --- | --- | --- | --- | --- | --- |
| MHI | .145 | | .062 | 2.33 | | .02 | .023 | | .267 | | ** |
| Constant | 44.164 | | 5.08 | 8.69 | | 0 | 34.208 | | 54.121 | | *** |
|  | | | | | | | | | | | |
| Mean dependent var | | 55.056 | | | SD dependent var | | | 24.339 | |  |  |
| Overall r-squared | | 0.016 | | | Number of obs | | | 208 | |  |  |
| Chi-square | | 5.446 | | | Prob > chi2 | | | 0.020 | |  |  |
| R-squared within | | 0.000 | | | R-squared between | | | 0.050 | |  |  |
| **** p<.01, ** p<.05, * p<.1, MHI median household income in US$ 1,000, child poverty rate in %* | | | | | | | | | | | |
|  | | | | | | | | | | | |

**Regression results**

| overall mission interval | Coef. | | St.Err. | t-value | | p-value | [95% Conf | | Interval] | | Sig |
| --- | --- | --- | --- | --- | --- | --- | --- | --- | --- | --- | --- |
| child poverty rate | -.039 | | .13 | -0.30 | | .763 | -.295 | | .216 | |  |
| Constant | 55.599 | | 2.823 | 19.70 | | 0 | 50.067 | | 61.132 | | *** |
|  | | | | | | | | | | | |
| Mean dependent var | | 55.056 | | | SD dependent var | | | 24.339 | |  |  |
| Overall r-squared | | 0.001 | | | Number of obs | | | 208 | |  |  |
| Chi-square | | 0.091 | | | Prob > chi2 | | | 0.763 | |  |  |
| R-squared within | | 0.000 | | | R-squared between | | | 0.002 | |  |  |
| **** p<.01, ** p<.05, * p<.1, MHI median household income in US$ 1,000, child poverty rate in %* | | | | | | | | | | | |
|  | | | | | | | | | | | |

**Regression results**

| overall mission interval | Coef. | | St.Err. | t-value | | p-value | [95% Conf | | Interval] | | Sig |
| --- | --- | --- | --- | --- | --- | --- | --- | --- | --- | --- | --- |
| COVID-19 | .977 | | 3.433 | 0.28 | | .776 | -5.752 | | 7.706 | |  |
| Constant | 54.564 | | 2.639 | 20.67 | | 0 | 49.391 | | 59.737 | | *** |
|  | | | | | | | | | | | |
| Mean dependent var | | 55.056 | | | SD dependent var | | | 24.339 | |  |  |
| Overall r-squared | | 0.001 | | | Number of obs | | | 208 | |  |  |
| Chi-square | | 0.081 | | | Prob > chi2 | | | 0.776 | |  |  |
| R-squared within | | 0.002 | | | R-squared between | | | 0.008 | |  |  |
| **** p<.01, ** p<.05, * p<.1, MHI median household income in US$ 1,000, child poverty rate in %* | | | | | | | | | | | |
|  | | | | | | | | | | | |

**Regression results**

| response interval (log) | Coef. | | St.Err. | t-value | | p-value | [95% Conf | | Interval] | | Sig |
| --- | --- | --- | --- | --- | --- | --- | --- | --- | --- | --- | --- |
| high-high cluster | -.264 | | .136 | -1.94 | | .053 | -.532 | | .003 | | * |
| Constant | 1.954 | | .047 | 41.53 | | 0 | 1.862 | | 2.046 | | *** |
|  | | | | | | | | | | | |
| Mean dependent var | | 1.927 | | | SD dependent var | | | 0.572 | |  |  |
| Overall r-squared | | 0.004 | | | Number of obs | | | 208 | |  |  |
| Chi-square | | 3.746 | | | Prob > chi2 | | | 0.053 | |  |  |
| R-squared within | | 0.000 | | | R-squared between | | | 0.056 | |  |  |
| **** p<.01, ** p<.05, * p<.1, MHI median household income in US$ 1,000, child poverty rate in %* | | | | | | | | | | | |
|  | | | | | | | | | | | |

**Regression results**

| on-scene interval | Coef. | | St.Err. | t-value | | p-value | [95% Conf | | Interval] | | Sig |
| --- | --- | --- | --- | --- | --- | --- | --- | --- | --- | --- | --- |
| high-high cluster | 5.537 | | 1.698 | 3.26 | | .001 | 2.208 | | 8.866 | | *** |
| Constant | 13.064 | | .667 | 19.58 | | 0 | 11.756 | | 14.371 | | *** |
|  | | | | | | | | | | | |
| Mean dependent var | | 13.918 | | | SD dependent var | | | 8.625 | |  |  |
| Overall r-squared | | 0.054 | | | Number of obs | | | 188 | |  |  |
| Chi-square | | 10.628 | | | Prob > chi2 | | | 0.001 | |  |  |
| R-squared within | | 0.000 | | | R-squared between | | | 0.039 | |  |  |
| **** p<.01, ** p<.05, * p<.1, MHI median household income in US$ 1,000, child poverty rate in %* | | | | | | | | | | | |
|  | | | | | | | | | | | |

**Regression results**

| transport interval (log) | Coef. | | St.Err. | t-value | | p-value | [95% Conf | | Interval] | | Sig |
| --- | --- | --- | --- | --- | --- | --- | --- | --- | --- | --- | --- |
| high-high cluster | -.462 | | .189 | -2.44 | | .015 | -.834 | | -.091 | | ** |
| Constant | 2.249 | | .061 | 37.12 | | 0 | 2.131 | | 2.368 | | *** |
|  | | | | | | | | | | | |
| Mean dependent var | | 2.185 | | | SD dependent var | | | 0.673 | |  |  |
| Overall r-squared | | 0.032 | | | Number of obs | | | 181 | |  |  |
| Chi-square | | 5.965 | | | Prob > chi2 | | | 0.015 | |  |  |
| R-squared within | | 0.000 | | | R-squared between | | | 0.052 | |  |  |
| **** p<.01, ** p<.05, * p<.1, MHI median household income in US$ 1,000, child poverty rate in %* | | | | | | | | | | | |
|  | | | | | | | | | | | |

**Regression results**

| back-to-service interval (log) | Coef. | | St.Err. | t-value | | p-value | [95% Conf | | Interval] | | Sig |
| --- | --- | --- | --- | --- | --- | --- | --- | --- | --- | --- | --- |
| high-high cluster | .182 | | .354 | 0.51 | | .607 | -.512 | | .875 | |  |
| Constant | 2.929 | | .11 | 26.71 | | 0 | 2.714 | | 3.144 | | *** |
|  | | | | | | | | | | | |
| Mean dependent var | | 2.985 | | | SD dependent var | | | 0.981 | |  |  |
| Overall r-squared | | 0.002 | | | Number of obs | | | 181 | |  |  |
| Chi-square | | 0.264 | | | Prob > chi2 | | | 0.607 | |  |  |
| R-squared within | | . | | | R-squared between | | | 0.003 | |  |  |
| **** p<.01, ** p<.05, * p<.1, MHI median household income in US$ 1,000, child poverty rate in %* | | | | | | | | | | | |
|  | | | | | | | | | | | |

**Regression results**

| overall mission interval | Coef. | | St.Err. | t-value | | p-value | [95% Conf | | Interval] | | Sig |
| --- | --- | --- | --- | --- | --- | --- | --- | --- | --- | --- | --- |
| high-high cluster | -3.067 | | 6.796 | -0.45 | | .652 | -16.388 | | 10.253 | |  |
| Constant | 55.35 | | 2.21 | 25.05 | | 0 | 51.019 | | 59.68 | | *** |
|  | | | | | | | | | | | |
| Mean dependent var | | 55.056 | | | SD dependent var | | | 24.339 | |  |  |
| Overall r-squared | | 0.003 | | | Number of obs | | | 208 | |  |  |
| Chi-square | | 0.204 | | | Prob > chi2 | | | 0.652 | |  |  |
| R-squared within | | 0.000 | | | R-squared between | | | 0.001 | |  |  |
| **** p<.01, ** p<.05, * p<.1, MHI median household income in US$ 1,000, child poverty rate in %* | | | | | | | | | | | |
|  | | | | | | | | | | | |

**Regression results**

| response interval (log) | Coef. | | St.Err. | t-value | | p-value | [95% Conf | | Interval] | | Sig |
| --- | --- | --- | --- | --- | --- | --- | --- | --- | --- | --- | --- |
| high-low outlier | -.156 | | .306 | -0.51 | | .61 | -.757 | | .444 | |  |
| Constant | 1.927 | | .046 | 41.90 | | 0 | 1.837 | | 2.017 | | *** |
|  | | | | | | | | | | | |
| Mean dependent var | | 1.927 | | | SD dependent var | | | 0.572 | |  |  |
| Overall r-squared | | 0.002 | | | Number of obs | | | 208 | |  |  |
| Chi-square | | 0.259 | | | Prob > chi2 | | | 0.610 | |  |  |
| R-squared within | | 0.000 | | | R-squared between | | | 0.002 | |  |  |
| **** p<.01, ** p<.05, * p<.1, MHI median household income in US$ 1,000, child poverty rate in %* | | | | | | | | | | | |
|  | | | | | | | | | | | |

**Regression results**

| on-scene interval | Coef. | | St.Err. | t-value | | p-value | [95% Conf | | Interval] | | Sig |
| --- | --- | --- | --- | --- | --- | --- | --- | --- | --- | --- | --- |
| high-low outlier | -6.33 | | 5.012 | -1.26 | | .207 | -16.153 | | 3.494 | |  |
| Constant | 14.019 | | .633 | 22.14 | | 0 | 12.778 | | 15.26 | | *** |
|  | | | | | | | | | | | |
| Mean dependent var | | 13.918 | | | SD dependent var | | | 8.625 | |  |  |
| Overall r-squared | | 0.009 | | | Number of obs | | | 188 | |  |  |
| Chi-square | | 1.595 | | | Prob > chi2 | | | 0.207 | |  |  |
| R-squared within | | 0.000 | | | R-squared between | | | 0.017 | |  |  |
| **** p<.01, ** p<.05, * p<.1, MHI median household income in US$ 1,000, child poverty rate in %* | | | | | | | | | | | |
|  | | | | | | | | | | | |

**Regression results**

| transport interval (log) | Coef. | | St.Err. | t-value | | p-value | [95% Conf | | Interval] | | Sig |
| --- | --- | --- | --- | --- | --- | --- | --- | --- | --- | --- | --- |
| high-low outlier | .279 | | .437 | 0.64 | | .523 | -.577 | | 1.135 | |  |
| Constant | 2.197 | | .059 | 36.93 | | 0 | 2.081 | | 2.314 | | *** |
|  | | | | | | | | | | | |
| Mean dependent var | | 2.185 | | | SD dependent var | | | 0.673 | |  |  |
| Overall r-squared | | 0.003 | | | Number of obs | | | 181 | |  |  |
| Chi-square | | 0.408 | | | Prob > chi2 | | | 0.523 | |  |  |
| R-squared within | | 0.000 | | | R-squared between | | | 0.003 | |  |  |
| **** p<.01, ** p<.05, * p<.1, MHI median household income in US$ 1,000, child poverty rate in %* | | | | | | | | | | | |
|  | | | | | | | | | | | |

**Regression results**

| back-to-service interval (log) | Coef. | | St.Err. | t-value | | p-value | [95% Conf | | Interval] | | Sig |
| --- | --- | --- | --- | --- | --- | --- | --- | --- | --- | --- | --- |
| high-low outlier | .106 | | .772 | 0.14 | | .89 | -1.407 | | 1.62 | |  |
| Constant | 2.944 | | .105 | 27.94 | | 0 | 2.738 | | 3.151 | | *** |
|  | | | | | | | | | | | |
| Mean dependent var | | 2.985 | | | SD dependent var | | | 0.981 | |  |  |
| Overall r-squared | | 0.000 | | | Number of obs | | | 181 | |  |  |
| Chi-square | | 0.019 | | | Prob > chi2 | | | 0.890 | |  |  |
| R-squared within | | 0.000 | | | R-squared between | | | 0.000 | |  |  |
| **** p<.01, ** p<.05, * p<.1, MHI median household income in US$ 1,000, child poverty rate in %* | | | | | | | | | | | |
|  | | | | | | | | | | | |

**Regression results**

| overall mission interval | Coef. | | St.Err. | t-value | | p-value | [95% Conf | | Interval] | | Sig |
| --- | --- | --- | --- | --- | --- | --- | --- | --- | --- | --- | --- |
| high-low outlier | -14.41 | | 13.664 | -1.05 | | .292 | -41.19 | | 12.371 | |  |
| Constant | 55.368 | | 2.103 | 26.32 | | 0 | 51.246 | | 59.49 | | *** |
|  | | | | | | | | | | | |
| Mean dependent var | | 55.056 | | | SD dependent var | | | 24.339 | |  |  |
| Overall r-squared | | 0.005 | | | Number of obs | | | 208 | |  |  |
| Chi-square | | 1.112 | | | Prob > chi2 | | | 0.292 | |  |  |
| R-squared within | | 0.000 | | | R-squared between | | | 0.012 | |  |  |
| **** p<.01, ** p<.05, * p<.1, MHI median household income in US$ 1,000, child poverty rate in %* | | | | | | | | | | | |
|  | | | | | | | | | | | |

**Regression results**

| response interval (log) | Coef. | | St.Err. | t-value | | p-value | [95% Conf | | Interval] | | Sig |
| --- | --- | --- | --- | --- | --- | --- | --- | --- | --- | --- | --- |
| low-high outlier | 1.217 | | .574 | 2.12 | | .034 | .093 | | 2.342 | | ** |
| Constant | 1.916 | | .044 | 43.11 | | 0 | 1.828 | | 2.003 | | *** |
|  | | | | | | | | | | | |
| Mean dependent var | | 1.927 | | | SD dependent var | | | 0.572 | |  |  |
| Overall r-squared | | 0.022 | | | Number of obs | | | 208 | |  |  |
| Chi-square | | 4.501 | | | Prob > chi2 | | | 0.034 | |  |  |
| R-squared within | | 0.000 | | | R-squared between | | | 0.045 | |  |  |
| **** p<.01, ** p<.05, * p<.1, MHI median household income in US$ 1,000, child poverty rate in %* | | | | | | | | | | | |
|  | | | | | | | | | | | |

**Regression results**

| on-scene interval | Coef. | | St.Err. | t-value | | p-value | [95% Conf | | Interval] | | Sig |
| --- | --- | --- | --- | --- | --- | --- | --- | --- | --- | --- | --- |
| low-high outlier | -2.062 | | 8.67 | -0.24 | | .812 | -19.056 | | 14.932 | |  |
| Constant | 13.929 | | .632 | 22.03 | | 0 | 12.689 | | 15.168 | | *** |
|  | | | | | | | | | | | |
| Mean dependent var | | 13.918 | | | SD dependent var | | | 8.625 | |  |  |
| Overall r-squared | | 0.000 | | | Number of obs | | | 188 | |  |  |
| Chi-square | | 0.057 | | | Prob > chi2 | | | 0.812 | |  |  |
| R-squared within | | 0.000 | | | R-squared between | | | 0.000 | |  |  |
| **** p<.01, ** p<.05, * p<.1, MHI median household income in US$ 1,000, child poverty rate in %* | | | | | | | | | | | |
|  | | | | | | | | | | | |

**Regression results**

| transport interval (log) | Coef. | | St.Err. | t-value | | p-value | [95% Conf | | Interval] | | Sig |
| --- | --- | --- | --- | --- | --- | --- | --- | --- | --- | --- | --- |
| low-high outlier | .809 | | .669 | 1.21 | | .227 | -.503 | | 2.12 | |  |
| Constant | 2.196 | | .059 | 37.33 | | 0 | 2.081 | | 2.311 | | *** |
|  | | | | | | | | | | | |
| Mean dependent var | | 2.185 | | | SD dependent var | | | 0.673 | |  |  |
| Overall r-squared | | 0.008 | | | Number of obs | | | 181 | |  |  |
| Chi-square | | 1.461 | | | Prob > chi2 | | | 0.227 | |  |  |
| R-squared within | | . | | | R-squared between | | | 0.016 | |  |  |
| **** p<.01, ** p<.05, * p<.1, MHI median household income in US$ 1,000, child poverty rate in %* | | | | | | | | | | | |
|  | | | | | | | | | | | |

**Regression results**

| back-to-service interval (log) | Coef. | | St.Err. | t-value | | p-value | [95% Conf | | Interval] | | Sig |
| --- | --- | --- | --- | --- | --- | --- | --- | --- | --- | --- | --- |
| low-high outlier | .414 | | 1.131 | 0.37 | | .715 | -1.804 | | 2.631 | |  |
| Constant | 2.943 | | .105 | 28.08 | | 0 | 2.737 | | 3.148 | | *** |
|  | | | | | | | | | | | |
| Mean dependent var | | 2.985 | | | SD dependent var | | | 0.981 | |  |  |
| Overall r-squared | | 0.001 | | | Number of obs | | | 181 | |  |  |
| Chi-square | | 0.134 | | | Prob > chi2 | | | 0.715 | |  |  |
| R-squared within | | 0.000 | | | R-squared between | | | 0.001 | |  |  |
| **** p<.01, ** p<.05, * p<.1, MHI median household income in US$ 1,000, child poverty rate in %* | | | | | | | | | | | |
|  | | | | | | | | | | | |

**Regression results**

| overall mission interval | Coef. | | St.Err. | t-value | | p-value | [95% Conf | | Interval] | | Sig |
| --- | --- | --- | --- | --- | --- | --- | --- | --- | --- | --- | --- |
| low-high outlier | 28.837 | | 25.21 | 1.14 | | .253 | -20.574 | | 78.248 | |  |
| Constant | 54.829 | | 2.084 | 26.32 | | 0 | 50.746 | | 58.913 | | *** |
|  | | | | | | | | | | | |
| Mean dependent var | | 55.056 | | | SD dependent var | | | 24.339 | |  |  |
| Overall r-squared | | 0.007 | | | Number of obs | | | 208 | |  |  |
| Chi-square | | 1.308 | | | Prob > chi2 | | | 0.253 | |  |  |
| R-squared within | | 0.000 | | | R-squared between | | | 0.013 | |  |  |
| **** p<.01, ** p<.05, * p<.1, MHI median household income in US$ 1,000, child poverty rate in %* | | | | | | | | | | | |
|  | | | | | | | | | | | |

**Regression results**

| response interval (log) | Coef. | | St.Err. | t-value | | p-value | [95% Conf | | Interval] | | Sig |
| --- | --- | --- | --- | --- | --- | --- | --- | --- | --- | --- | --- |
| low-low cluster | .031 | | .197 | 0.16 | | .874 | -.355 | | .418 | |  |
| Constant | 1.922 | | .047 | 41.02 | | 0 | 1.83 | | 2.014 | | *** |
|  | | | | | | | | | | | |
| Mean dependent var | | 1.927 | | | SD dependent var | | | 0.572 | |  |  |
| Overall r-squared | | 0.000 | | | Number of obs | | | 208 | |  |  |
| Chi-square | | 0.025 | | | Prob > chi2 | | | 0.874 | |  |  |
| R-squared within | | 0.000 | | | R-squared between | | | 0.001 | |  |  |
| **** p<.01, ** p<.05, * p<.1, MHI median household income in US$ 1,000, child poverty rate in %* | | | | | | | | | | | |
|  | | | | | | | | | | | |

**Regression results**

| on-scene interval | Coef. | | St.Err. | t-value | | p-value | [95% Conf | | Interval] | | Sig |
| --- | --- | --- | --- | --- | --- | --- | --- | --- | --- | --- | --- |
| low-low cluster | -2.365 | | 2.682 | -0.88 | | .378 | -7.621 | | 2.891 | |  |
| Constant | 14.056 | | .649 | 21.67 | | 0 | 12.785 | | 15.327 | | *** |
|  | | | | | | | | | | | |
| Mean dependent var | | 13.918 | | | SD dependent var | | | 8.625 | |  |  |
| Overall r-squared | | 0.004 | | | Number of obs | | | 188 | |  |  |
| Chi-square | | 0.778 | | | Prob > chi2 | | | 0.378 | |  |  |
| R-squared within | | 0.000 | | | R-squared between | | | 0.003 | |  |  |
| **** p<.01, ** p<.05, * p<.1, MHI median household income in US$ 1,000, child poverty rate in %* | | | | | | | | | | | |
|  | | | | | | | | | | | |

**Regression results**

| transport interval (log) | Coef. | | St.Err. | t-value | | p-value | [95% Conf | | Interval] | | Sig |
| --- | --- | --- | --- | --- | --- | --- | --- | --- | --- | --- | --- |
| low-low cluster | -.107 | | .243 | -0.44 | | .66 | -.583 | | .369 | |  |
| Constant | 2.209 | | .061 | 36.24 | | 0 | 2.09 | | 2.329 | | *** |
|  | | | | | | | | | | | |
| Mean dependent var | | 2.185 | | | SD dependent var | | | 0.673 | |  |  |
| Overall r-squared | | 0.006 | | | Number of obs | | | 181 | |  |  |
| Chi-square | | 0.194 | | | Prob > chi2 | | | 0.660 | |  |  |
| R-squared within | | 0.000 | | | R-squared between | | | 0.000 | |  |  |
| **** p<.01, ** p<.05, * p<.1, MHI median household income in US$ 1,000, child poverty rate in %* | | | | | | | | | | | |
|  | | | | | | | | | | | |

**Regression results**

| back-to-service interval (log) | Coef. | | St.Err. | t-value | | p-value | [95% Conf | | Interval] | | Sig |
| --- | --- | --- | --- | --- | --- | --- | --- | --- | --- | --- | --- |
| low-low cluster | .37 | | .425 | 0.87 | | .384 | -.463 | | 1.204 | |  |
| Constant | 2.923 | | .108 | 27.18 | | 0 | 2.712 | | 3.133 | | *** |
|  | | | | | | | | | | | |
| Mean dependent var | | 2.985 | | | SD dependent var | | | 0.981 | |  |  |
| Overall r-squared | | 0.009 | | | Number of obs | | | 181 | |  |  |
| Chi-square | | 0.758 | | | Prob > chi2 | | | 0.384 | |  |  |
| R-squared within | | 0.000 | | | R-squared between | | | 0.007 | |  |  |
| **** p<.01, ** p<.05, * p<.1, MHI median household income in US$ 1,000, child poverty rate in %* | | | | | | | | | | | |
|  | | | | | | | | | | | |

**Regression results**

| overall mission interval | Coef. | | St.Err. | t-value | | p-value | [95% Conf | | Interval] | | Sig |
| --- | --- | --- | --- | --- | --- | --- | --- | --- | --- | --- | --- |
| low-low cluster | 6.159 | | 8.976 | 0.69 | | .493 | -11.433 | | 23.751 | |  |
| Constant | 54.673 | | 2.149 | 25.45 | | 0 | 50.462 | | 58.884 | | *** |
|  | | | | | | | | | | | |
| Mean dependent var | | 55.056 | | | SD dependent var | | | 24.339 | |  |  |
| Overall r-squared | | 0.004 | | | Number of obs | | | 208 | |  |  |
| Chi-square | | 0.471 | | | Prob > chi2 | | | 0.493 | |  |  |
| R-squared within | | 0.000 | | | R-squared between | | | 0.004 | |  |  |
| **** p<.01, ** p<.05, * p<.1, MHI median household income in US$ 1,000, child poverty rate in %* | | | | | | | | | | | |
|  | | | | | | | | | | | |

**Regression results**

| response interval (log) | Coef. | | St.Err. | t-value | | p-value | [95% Conf | | Interval] | | Sig |
| --- | --- | --- | --- | --- | --- | --- | --- | --- | --- | --- | --- |
| MHI | .004 | | .001 | 2.92 | | .004 | .001 | | .006 | | *** |
| Constant | 1.643 | | .105 | 15.61 | | 0 | 1.436 | | 1.849 | | *** |
|  | | | | | | | | | | | |
| Mean dependent var | | 1.927 | | | SD dependent var | | | 0.572 | |  |  |
| Overall r-squared | | 0.048 | | | Number of obs | | | 208 | |  |  |
| Chi-square | | 8.505 | | | Prob > chi2 | | | 0.004 | |  |  |
| R-squared within | | 0.000 | | | R-squared between | | | 0.076 | |  |  |
| **** p<.01, ** p<.05, * p<.1, MHI median household income in US$ 1,000, child poverty rate in %* | | | | | | | | | | | |
|  | | | | | | | | | | | |

**Regression results**

| response interval (log) | Coef. | | St.Err. | t-value | | p-value | [95% Conf | | Interval] | | Sig |
| --- | --- | --- | --- | --- | --- | --- | --- | --- | --- | --- | --- |
| child poverty rate | -.003 | | .003 | -0.94 | | .349 | -.008 | | .003 | |  |
| Constant | 1.962 | | .061 | 32.11 | | 0 | 1.842 | | 2.082 | | *** |
|  | | | | | | | | | | | |
| Mean dependent var | | 1.927 | | | SD dependent var | | | 0.572 | |  |  |
| Overall r-squared | | 0.005 | | | Number of obs | | | 208 | |  |  |
| Chi-square | | 0.878 | | | Prob > chi2 | | | 0.349 | |  |  |
| R-squared within | | 0.000 | | | R-squared between | | | 0.011 | |  |  |
| **** p<.01, ** p<.05, * p<.1, MHI median household income in US$ 1,000, child poverty rate in %* | | | | | | | | | | | |
|  | | | | | | | | | | | |

**Regression results**

| response interval (log) | Coef. | | St.Err. | t-value | | p-value | [95% Conf | | Interval] | | Sig |
| --- | --- | --- | --- | --- | --- | --- | --- | --- | --- | --- | --- |
| COVID-19 | -.051 | | .082 | -0.62 | | .535 | -.211 | | .11 | |  |
| Constant | 1.947 | | .059 | 32.75 | | 0 | 1.831 | | 2.064 | | *** |
|  | | | | | | | | | | | |
| Mean dependent var | | 1.927 | | | SD dependent var | | | 0.572 | |  |  |
| Overall r-squared | | 0.003 | | | Number of obs | | | 208 | |  |  |
| Chi-square | | 0.385 | | | Prob > chi2 | | | 0.535 | |  |  |
| R-squared within | | 0.002 | | | R-squared between | | | 0.001 | |  |  |
| **** p<.01, ** p<.05, * p<.1, MHI median household income in US$ 1,000, child poverty rate in %* | | | | | | | | | | | |
|  | | | | | | | | | | | |

**Regression results**

| on-scene interval | Coef. | | St.Err. | t-value | | p-value | [95% Conf | | Interval] | | Sig |
| --- | --- | --- | --- | --- | --- | --- | --- | --- | --- | --- | --- |
| MHI | .057 | | .017 | 3.46 | | .001 | .025 | | .09 | | *** |
| Constant | 9.49 | | 1.418 | 6.70 | | 0 | 6.712 | | 12.269 | | *** |
|  | | | | | | | | | | | |
| Mean dependent var | | 13.918 | | | SD dependent var | | | 8.625 | |  |  |
| Overall r-squared | | 0.061 | | | Number of obs | | | 188 | |  |  |
| Chi-square | | 11.983 | | | Prob > chi2 | | | 0.001 | |  |  |
| R-squared within | | 0.000 | | | R-squared between | | | 0.014 | |  |  |
| **** p<.01, ** p<.05, * p<.1, MHI median household income in US$ 1,000, child poverty rate in %* | | | | | | | | | | | |
|  | | | | | | | | | | | |

**Regression results**

| on-scene interval | Coef. | | St.Err. | t-value | | p-value | [95% Conf | | Interval] | | Sig |
| --- | --- | --- | --- | --- | --- | --- | --- | --- | --- | --- | --- |
| child poverty rate | -.028 | | .037 | -0.75 | | .455 | -.1 | | .045 | |  |
| Constant | 14.342 | | .848 | 16.92 | | 0 | 12.68 | | 16.003 | | *** |
|  | | | | | | | | | | | |
| Mean dependent var | | 13.918 | | | SD dependent var | | | 8.625 | |  |  |
| Overall r-squared | | 0.003 | | | Number of obs | | | 188 | |  |  |
| Chi-square | | 0.558 | | | Prob > chi2 | | | 0.455 | |  |  |
| R-squared within | | 0.000 | | | R-squared between | | | 0.012 | |  |  |
| **** p<.01, ** p<.05, * p<.1, MHI median household income in US$ 1,000, child poverty rate in %* | | | | | | | | | | | |
|  | | | | | | | | | | | |

**Regression results**

| on-scene interval | Coef. | | St.Err. | t-value | | p-value | [95% Conf | | Interval] | | Sig |
| --- | --- | --- | --- | --- | --- | --- | --- | --- | --- | --- | --- |
| COVID-19 | -3.625 | | 1.238 | -2.93 | | .003 | -6.051 | | -1.199 | | *** |
| Constant | 15.576 | | .837 | 18.60 | | 0 | 13.935 | | 17.217 | | *** |
|  | | | | | | | | | | | |
| Mean dependent var | | 13.918 | | | SD dependent var | | | 8.625 | |  |  |
| Overall r-squared | | 0.044 | | | Number of obs | | | 188 | |  |  |
| Chi-square | | 8.574 | | | Prob > chi2 | | | 0.003 | |  |  |
| R-squared within | | 0.041 | | | R-squared between | | | 0.025 | |  |  |
| **** p<.01, ** p<.05, * p<.1, MHI median household income in US$ 1,000, child poverty rate in %* | | | | | | | | | | | |
|  | | | | | | | | | | | |

**Regression results**

| transport interval (log) | Coef. | | St.Err. | t-value | | p-value | [95% Conf | | Interval] | | Sig |
| --- | --- | --- | --- | --- | --- | --- | --- | --- | --- | --- | --- |
| MHI | .008 | | .002 | 5.24 | | 0 | .005 | | .011 | | *** |
| Constant | 1.581 | | .129 | 12.25 | | 0 | 1.328 | | 1.834 | | *** |
|  | | | | | | | | | | | |
| Mean dependent var | | 2.185 | | | SD dependent var | | | 0.673 | |  |  |
| Overall r-squared | | 0.145 | | | Number of obs | | | 181 | |  |  |
| Chi-square | | 27.437 | | | Prob > chi2 | | | 0.000 | |  |  |
| R-squared within | | 0.000 | | | R-squared between | | | 0.210 | |  |  |
| **** p<.01, ** p<.05, * p<.1, MHI median household income in US$ 1,000, child poverty rate in %* | | | | | | | | | | | |
|  | | | | | | | | | | | |

**Regression results**

| transport interval (log) | Coef. | | St.Err. | t-value | | p-value | [95% Conf | | Interval] | | Sig |
| --- | --- | --- | --- | --- | --- | --- | --- | --- | --- | --- | --- |
| child poverty rate | -.013 | | .003 | -3.73 | | 0 | -.019 | | -.006 | | *** |
| Constant | 2.389 | | .075 | 31.97 | | 0 | 2.242 | | 2.535 | | *** |
|  | | | | | | | | | | | |
| Mean dependent var | | 2.185 | | | SD dependent var | | | 0.673 | |  |  |
| Overall r-squared | | 0.080 | | | Number of obs | | | 181 | |  |  |
| Chi-square | | 13.929 | | | Prob > chi2 | | | 0.000 | |  |  |
| R-squared within | | 0.000 | | | R-squared between | | | 0.117 | |  |  |
| **** p<.01, ** p<.05, * p<.1, MHI median household income in US$ 1,000, child poverty rate in %* | | | | | | | | | | | |
|  | | | | | | | | | | | |

**Regression results**

| transport interval (log) | Coef. | | St.Err. | t-value | | p-value | [95% Conf | | Interval] | | Sig |
| --- | --- | --- | --- | --- | --- | --- | --- | --- | --- | --- | --- |
| COVID-19 | .239 | | .092 | 2.61 | | .009 | .059 | | .419 | | *** |
| Constant | 2.087 | | .073 | 28.43 | | 0 | 1.943 | | 2.231 | | *** |
|  | | | | | | | | | | | |
| Mean dependent var | | 2.185 | | | SD dependent var | | | 0.673 | |  |  |
| Overall r-squared | | 0.019 | | | Number of obs | | | 181 | |  |  |
| Chi-square | | 6.794 | | | Prob > chi2 | | | 0.009 | |  |  |
| R-squared within | | 0.069 | | | R-squared between | | | 0.022 | |  |  |
| **** p<.01, ** p<.05, * p<.1, MHI median household income in US$ 1,000, child poverty rate in %* | | | | | | | | | | | |
|  | | | | | | | | | | | |

**Regression results**

| back-to-service interval (log) | Coef. | | St.Err. | t-value | | p-value | [95% Conf | | Interval] | | Sig |
| --- | --- | --- | --- | --- | --- | --- | --- | --- | --- | --- | --- |
| MHI | -.001 | | .003 | -0.31 | | .756 | -.007 | | .005 | |  |
| Constant | 3.022 | | .265 | 11.38 | | 0 | 2.502 | | 3.542 | | *** |
|  | | | | | | | | | | | |
| Mean dependent var | | 2.985 | | | SD dependent var | | | 0.981 | |  |  |
| Overall r-squared | | 0.000 | | | Number of obs | | | 181 | |  |  |
| Chi-square | | 0.096 | | | Prob > chi2 | | | 0.756 | |  |  |
| R-squared within | | 0.000 | | | R-squared between | | | 0.001 | |  |  |
| **** p<.01, ** p<.05, * p<.1, MHI median household income in US$ 1,000, child poverty rate in %* | | | | | | | | | | | |
|  | | | | | | | | | | | |

**Regression results**

| back-to-service interval (log) | Coef. | | St.Err. | t-value | | p-value | [95% Conf | | Interval] | | Sig |
| --- | --- | --- | --- | --- | --- | --- | --- | --- | --- | --- | --- |
| child poverty rate | .002 | | .007 | 0.32 | | .747 | -.011 | | .015 | |  |
| Constant | 2.916 | | .141 | 20.72 | | 0 | 2.64 | | 3.192 | | *** |
|  | | | | | | | | | | | |
| Mean dependent var | | 2.985 | | | SD dependent var | | | 0.981 | |  |  |
| Overall r-squared | | 0.010 | | | Number of obs | | | 181 | |  |  |
| Chi-square | | 0.104 | | | Prob > chi2 | | | 0.747 | |  |  |
| R-squared within | | 0.000 | | | R-squared between | | | 0.000 | |  |  |
| **** p<.01, ** p<.05, * p<.1, MHI median household income in US$ 1,000, child poverty rate in %* | | | | | | | | | | | |
|  | | | | | | | | | | | |

**Regression results**

| back-to-service interval (log) | Coef. | | St.Err. | t-value | | p-value | [95% Conf | | Interval] | | Sig |
| --- | --- | --- | --- | --- | --- | --- | --- | --- | --- | --- | --- |
| COVID-19 | .117 | | .13 | 0.90 | | .368 | -.138 | | .372 | |  |
| Constant | 2.889 | | .122 | 23.67 | | 0 | 2.65 | | 3.128 | | *** |
|  | | | | | | | | | | | |
| Mean dependent var | | 2.985 | | | SD dependent var | | | 0.981 | |  |  |
| Overall r-squared | | 0.000 | | | Number of obs | | | 181 | |  |  |
| Chi-square | | 0.810 | | | Prob > chi2 | | | 0.368 | |  |  |
| R-squared within | | 0.021 | | | R-squared between | | | 0.001 | |  |  |
| **** p<.01, ** p<.05, * p<.1, MHI median household income in US$ 1,000, child poverty rate in %* | | | | | | | | | | | |
|  | | | | | | | | | | | |

**Regression results**

| overall mission interval | Coef. | | St.Err. | t-value | | p-value | [95% Conf | | Interval] | | Sig |
| --- | --- | --- | --- | --- | --- | --- | --- | --- | --- | --- | --- |
| MHI | .145 | | .062 | 2.33 | | .02 | .023 | | .267 | | ** |
| Constant | 44.164 | | 5.08 | 8.69 | | 0 | 34.208 | | 54.121 | | *** |
|  | | | | | | | | | | | |
| Mean dependent var | | 55.056 | | | SD dependent var | | | 24.339 | |  |  |
| Overall r-squared | | 0.016 | | | Number of obs | | | 208 | |  |  |
| Chi-square | | 5.446 | | | Prob > chi2 | | | 0.020 | |  |  |
| R-squared within | | 0.000 | | | R-squared between | | | 0.050 | |  |  |
| **** p<.01, ** p<.05, * p<.1, MHI median household income in US$ 1,000, child poverty rate in %* | | | | | | | | | | | |
|  | | | | | | | | | | | |

**Regression results**

| overall mission interval | Coef. | | St.Err. | t-value | | p-value | [95% Conf | | Interval] | | Sig |
| --- | --- | --- | --- | --- | --- | --- | --- | --- | --- | --- | --- |
| child poverty rate | -.039 | | .13 | -0.30 | | .763 | -.295 | | .216 | |  |
| Constant | 55.599 | | 2.823 | 19.70 | | 0 | 50.067 | | 61.132 | | *** |
|  | | | | | | | | | | | |
| Mean dependent var | | 55.056 | | | SD dependent var | | | 24.339 | |  |  |
| Overall r-squared | | 0.001 | | | Number of obs | | | 208 | |  |  |
| Chi-square | | 0.091 | | | Prob > chi2 | | | 0.763 | |  |  |
| R-squared within | | 0.000 | | | R-squared between | | | 0.002 | |  |  |
| **** p<.01, ** p<.05, * p<.1, MHI median household income in US$ 1,000, child poverty rate in %* | | | | | | | | | | | |
|  | | | | | | | | | | | |

**Regression results**

| overall mission interval | Coef. | | St.Err. | t-value | | p-value | [95% Conf | | Interval] | | Sig |
| --- | --- | --- | --- | --- | --- | --- | --- | --- | --- | --- | --- |
| COVID-19 | .977 | | 3.433 | 0.28 | | .776 | -5.752 | | 7.706 | |  |
| Constant | 54.564 | | 2.639 | 20.67 | | 0 | 49.391 | | 59.737 | | *** |
|  | | | | | | | | | | | |
| Mean dependent var | | 55.056 | | | SD dependent var | | | 24.339 | |  |  |
| Overall r-squared | | 0.001 | | | Number of obs | | | 208 | |  |  |
| Chi-square | | 0.081 | | | Prob > chi2 | | | 0.776 | |  |  |
| R-squared within | | 0.002 | | | R-squared between | | | 0.008 | |  |  |
| **** p<.01, ** p<.05, * p<.1, MHI median household income in US$ 1,000, child poverty rate in %* | | | | | | | | | | | |
|  | | | | | | | | | | | |

**Regression results**

| response interval (log) | Coef. | | St.Err. | t-value | | p-value | [95% Conf | | Interval] | | Sig |
| --- | --- | --- | --- | --- | --- | --- | --- | --- | --- | --- | --- |
| high-high cluster | .091 | | .149 | 0.61 | | .539 | -.2 | | .383 | |  |
| Constant | 1.915 | | .047 | 40.41 | | 0 | 1.822 | | 2.008 | | *** |
|  | | | | | | | | | | | |
| Mean dependent var | | 1.927 | | | SD dependent var | | | 0.572 | |  |  |
| Overall r-squared | | 0.007 | | | Number of obs | | | 208 | |  |  |
| Chi-square | | 0.378 | | | Prob > chi2 | | | 0.539 | |  |  |
| R-squared within | | 0.002 | | | R-squared between | | | 0.002 | |  |  |
| **** p<.01, ** p<.05, * p<.1, MHI median household income in US$ 1,000, child poverty rate in %* | | | | | | | | | | | |
|  | | | | | | | | | | | |

**Regression results**

| on-scene interval | Coef. | | St.Err. | t-value | | p-value | [95% Conf | | Interval] | | Sig |
| --- | --- | --- | --- | --- | --- | --- | --- | --- | --- | --- | --- |
| high-high cluster | 6.579 | | 1.794 | 3.67 | | 0 | 3.063 | | 10.095 | | *** |
| Constant | 13.043 | | .654 | 19.94 | | 0 | 11.761 | | 14.325 | | *** |
|  | | | | | | | | | | | |
| Mean dependent var | | 13.918 | | | SD dependent var | | | 8.625 | |  |  |
| Overall r-squared | | 0.067 | | | Number of obs | | | 188 | |  |  |
| Chi-square | | 13.451 | | | Prob > chi2 | | | 0.000 | |  |  |
| R-squared within | | 0.022 | | | R-squared between | | | 0.035 | |  |  |
| **** p<.01, ** p<.05, * p<.1, MHI median household income in US$ 1,000, child poverty rate in %* | | | | | | | | | | | |
|  | | | | | | | | | | | |

**Regression results**

| transport interval (log) | Coef. | | St.Err. | t-value | | p-value | [95% Conf | | Interval] | | Sig |
| --- | --- | --- | --- | --- | --- | --- | --- | --- | --- | --- | --- |
| high-high cluster | -.177 | | .184 | -0.96 | | .337 | -.537 | | .184 | |  |
| Constant | 2.217 | | .061 | 36.58 | | 0 | 2.099 | | 2.336 | | *** |
|  | | | | | | | | | | | |
| Mean dependent var | | 2.185 | | | SD dependent var | | | 0.673 | |  |  |
| Overall r-squared | | 0.007 | | | Number of obs | | | 181 | |  |  |
| Chi-square | | 0.920 | | | Prob > chi2 | | | 0.337 | |  |  |
| R-squared within | | 0.001 | | | R-squared between | | | 0.016 | |  |  |
| **** p<.01, ** p<.05, * p<.1, MHI median household income in US$ 1,000, child poverty rate in %* | | | | | | | | | | | |
|  | | | | | | | | | | | |

**Regression results**

| back-to-service interval (log) | Coef. | | St.Err. | t-value | | p-value | [95% Conf | | Interval] | | Sig |
| --- | --- | --- | --- | --- | --- | --- | --- | --- | --- | --- | --- |
| high-high cluster | .088 | | .283 | 0.31 | | .755 | -.466 | | .643 | |  |
| Constant | 2.939 | | .106 | 27.61 | | 0 | 2.73 | | 3.148 | | *** |
|  | | | | | | | | | | | |
| Mean dependent var | | 2.985 | | | SD dependent var | | | 0.981 | |  |  |
| Overall r-squared | | 0.003 | | | Number of obs | | | 181 | |  |  |
| Chi-square | | 0.098 | | | Prob > chi2 | | | 0.755 | |  |  |
| R-squared within | | 0.005 | | | R-squared between | | | 0.011 | |  |  |
| **** p<.01, ** p<.05, * p<.1, MHI median household income in US$ 1,000, child poverty rate in %* | | | | | | | | | | | |
|  | | | | | | | | | | | |

**Regression results**

| overall mission interval | Coef. | | St.Err. | t-value | | p-value | [95% Conf | | Interval] | | Sig |
| --- | --- | --- | --- | --- | --- | --- | --- | --- | --- | --- | --- |
| high-high cluster | 10.799 | | 6.691 | 1.61 | | .107 | -2.315 | | 23.913 | |  |
| Constant | 54.111 | | 2.121 | 25.52 | | 0 | 49.955 | | 58.267 | | *** |
|  | | | | | | | | | | | |
| Mean dependent var | | 55.056 | | | SD dependent var | | | 24.339 | |  |  |
| Overall r-squared | | 0.002 | | | Number of obs | | | 208 | |  |  |
| Chi-square | | 2.605 | | | Prob > chi2 | | | 0.107 | |  |  |
| R-squared within | | 0.001 | | | R-squared between | | | 0.047 | |  |  |
| **** p<.01, ** p<.05, * p<.1, MHI median household income in US$ 1,000, child poverty rate in %* | | | | | | | | | | | |
|  | | | | | | | | | | | |

**Regression results**

| response interval (log) | Coef. | | St.Err. | t-value | | p-value | [95% Conf | | Interval] | | Sig |
| --- | --- | --- | --- | --- | --- | --- | --- | --- | --- | --- | --- |
| high-low outlier | -.088 | | .153 | -0.57 | | .567 | -.388 | | .212 | |  |
| Constant | 1.931 | | .047 | 40.94 | | 0 | 1.838 | | 2.023 | | *** |
|  | | | | | | | | | | | |
| Mean dependent var | | 1.927 | | | SD dependent var | | | 0.572 | |  |  |
| Overall r-squared | | 0.004 | | | Number of obs | | | 208 | |  |  |
| Chi-square | | 0.328 | | | Prob > chi2 | | | 0.567 | |  |  |
| R-squared within | | 0.009 | | | R-squared between | | | 0.009 | |  |  |
| **** p<.01, ** p<.05, * p<.1, MHI median household income in US$ 1,000, child poverty rate in %* | | | | | | | | | | | |
|  | | | | | | | | | | | |

**Regression results**

| on-scene interval | Coef. | | St.Err. | t-value | | p-value | [95% Conf | | Interval] | | Sig |
| --- | --- | --- | --- | --- | --- | --- | --- | --- | --- | --- | --- |
| high-low outlier | -2.287 | | 2.254 | -1.01 | | .31 | -6.706 | | 2.131 | |  |
| Constant | 14.112 | | .658 | 21.46 | | 0 | 12.823 | | 15.401 | | *** |
|  | | | | | | | | | | | |
| Mean dependent var | | 13.918 | | | SD dependent var | | | 8.625 | |  |  |
| Overall r-squared | | 0.006 | | | Number of obs | | | 188 | |  |  |
| Chi-square | | 1.030 | | | Prob > chi2 | | | 0.310 | |  |  |
| R-squared within | | 0.000 | | | R-squared between | | | 0.013 | |  |  |
| **** p<.01, ** p<.05, * p<.1, MHI median household income in US$ 1,000, child poverty rate in %* | | | | | | | | | | | |
|  | | | | | | | | | | | |

**Regression results**

| transport interval (log) | Coef. | | St.Err. | t-value | | p-value | [95% Conf | | Interval] | | Sig |
| --- | --- | --- | --- | --- | --- | --- | --- | --- | --- | --- | --- |
| high-low outlier | .089 | | .183 | 0.49 | | .626 | -.27 | | .448 | |  |
| Constant | 2.195 | | .061 | 36.08 | | 0 | 2.076 | | 2.314 | | *** |
|  | | | | | | | | | | | |
| Mean dependent var | | 2.185 | | | SD dependent var | | | 0.673 | |  |  |
| Overall r-squared | | 0.000 | | | Number of obs | | | 181 | |  |  |
| Chi-square | | 0.237 | | | Prob > chi2 | | | 0.626 | |  |  |
| R-squared within | | 0.006 | | | R-squared between | | | 0.000 | |  |  |
| **** p<.01, ** p<.05, * p<.1, MHI median household income in US$ 1,000, child poverty rate in %* | | | | | | | | | | | |
|  | | | | | | | | | | | |

**Regression results**

| back-to-service interval (log) | Coef. | | St.Err. | t-value | | p-value | [95% Conf | | Interval] | | Sig |
| --- | --- | --- | --- | --- | --- | --- | --- | --- | --- | --- | --- |
| high-low outlier | -.176 | | .27 | -0.65 | | .515 | -.705 | | .353 | |  |
| Constant | 2.961 | | .107 | 27.79 | | 0 | 2.752 | | 3.17 | | *** |
|  | | | | | | | | | | | |
| Mean dependent var | | 2.985 | | | SD dependent var | | | 0.981 | |  |  |
| Overall r-squared | | 0.000 | | | Number of obs | | | 181 | |  |  |
| Chi-square | | 0.425 | | | Prob > chi2 | | | 0.515 | |  |  |
| R-squared within | | 0.007 | | | R-squared between | | | 0.001 | |  |  |
| **** p<.01, ** p<.05, * p<.1, MHI median household income in US$ 1,000, child poverty rate in %* | | | | | | | | | | | |
|  | | | | | | | | | | | |

**Regression results**

| overall mission interval | Coef. | | St.Err. | t-value | | p-value | [95% Conf | | Interval] | | Sig |
| --- | --- | --- | --- | --- | --- | --- | --- | --- | --- | --- | --- |
| high-low outlier | -8.541 | | 6.625 | -1.29 | | .197 | -21.525 | | 4.443 | |  |
| Constant | 55.759 | | 2.148 | 25.95 | | 0 | 51.548 | | 59.97 | | *** |
|  | | | | | | | | | | | |
| Mean dependent var | | 55.056 | | | SD dependent var | | | 24.339 | |  |  |
| Overall r-squared | | 0.002 | | | Number of obs | | | 208 | |  |  |
| Chi-square | | 1.662 | | | Prob > chi2 | | | 0.197 | |  |  |
| R-squared within | | 0.008 | | | R-squared between | | | 0.014 | |  |  |
| **** p<.01, ** p<.05, * p<.1, MHI median household income in US$ 1,000, child poverty rate in %* | | | | | | | | | | | |
|  | | | | | | | | | | | |

**Regression results**

| response interval (log) | Coef. | | St.Err. | t-value | | p-value | [95% Conf | | Interval] | | Sig |
| --- | --- | --- | --- | --- | --- | --- | --- | --- | --- | --- | --- |
| LH | 0 | | . | . | | . | . | | . | |  |
| Constant | 1.923 | | .045 | 42.47 | | 0 | 1.835 | | 2.012 | | *** |
|  | | | | | | | | | | | |
| Mean dependent var | | 1.927 | | | SD dependent var | | | 0.572 | |  |  |
| Overall r-squared | | 0.000 | | | Number of obs | | | 208 | |  |  |
| Chi-square | | . | | | Prob > chi2 | | | . | |  |  |
| R-squared within | | 0.000 | | | R-squared between | | | 0.000 | |  |  |
| **** p<.01, ** p<.05, * p<.1, MHI median household income in US$ 1,000, child poverty rate in %* | | | | | | | | | | | |
|  | | | | | | | | | | | |

**Regression results**

| on-scene interval | Coef. | | St.Err. | t-value | | p-value | [95% Conf | | Interval] | | Sig |
| --- | --- | --- | --- | --- | --- | --- | --- | --- | --- | --- | --- |
| LH | 0 | | . | . | | . | . | | . | |  |
| Constant | 13.918 | | .629 | 22.12 | | 0 | 12.685 | | 15.151 | | *** |
|  | | | | | | | | | | | |
| Mean dependent var | | 13.918 | | | SD dependent var | | | 8.625 | |  |  |
| Overall r-squared | | 0.000 | | | Number of obs | | | 188 | |  |  |
| Chi-square | | . | | | Prob > chi2 | | | . | |  |  |
| R-squared within | | 0.000 | | | R-squared between | | | 0.000 | |  |  |
| **** p<.01, ** p<.05, * p<.1, MHI median household income in US$ 1,000, child poverty rate in %* | | | | | | | | | | | |
|  | | | | | | | | | | | |

**Regression results**

| transport interval (log) | Coef. | | St.Err. | t-value | | p-value | [95% Conf | | Interval] | | Sig |
| --- | --- | --- | --- | --- | --- | --- | --- | --- | --- | --- | --- |
| LH | 0 | | . | . | | . | . | | . | |  |
| Constant | 2.202 | | .059 | 37.48 | | 0 | 2.087 | | 2.318 | | *** |
|  | | | | | | | | | | | |
| Mean dependent var | | 2.185 | | | SD dependent var | | | 0.673 | |  |  |
| Overall r-squared | | 0.000 | | | Number of obs | | | 181 | |  |  |
| Chi-square | | . | | | Prob > chi2 | | | . | |  |  |
| R-squared within | | 0.000 | | | R-squared between | | | 0.000 | |  |  |
| **** p<.01, ** p<.05, * p<.1, MHI median household income in US$ 1,000, child poverty rate in %* | | | | | | | | | | | |
|  | | | | | | | | | | | |

**Regression results**

| back-to-service interval (log) | Coef. | | St.Err. | t-value | | p-value | [95% Conf | | Interval] | | Sig |
| --- | --- | --- | --- | --- | --- | --- | --- | --- | --- | --- | --- |
| LH | 0 | | . | . | | . | . | | . | |  |
| Constant | 2.946 | | .104 | 28.36 | | 0 | 2.743 | | 3.15 | | *** |
|  | | | | | | | | | | | |
| Mean dependent var | | 2.985 | | | SD dependent var | | | 0.981 | |  |  |
| Overall r-squared | | 0.000 | | | Number of obs | | | 181 | |  |  |
| Chi-square | | . | | | Prob > chi2 | | | . | |  |  |
| R-squared within | | 0.000 | | | R-squared between | | | 0.000 | |  |  |
| **** p<.01, ** p<.05, * p<.1, MHI median household income in US$ 1,000, child poverty rate in %* | | | | | | | | | | | |
|  | | | | | | | | | | | |

**Regression results**

| overall mission interval | Coef. | | St.Err. | t-value | | p-value | [95% Conf | | Interval] | | Sig |
| --- | --- | --- | --- | --- | --- | --- | --- | --- | --- | --- | --- |
| LH | 0 | | . | . | | . | . | | . | |  |
| Constant | 55.026 | | 2.081 | 26.45 | | 0 | 50.948 | | 59.104 | | *** |
|  | | | | | | | | | | | |
| Mean dependent var | | 55.056 | | | SD dependent var | | | 24.339 | |  |  |
| Overall r-squared | | 0.000 | | | Number of obs | | | 208 | |  |  |
| Chi-square | | . | | | Prob > chi2 | | | . | |  |  |
| R-squared within | | 0.000 | | | R-squared between | | | 0.000 | |  |  |
| **** p<.01, ** p<.05, * p<.1, MHI median household income in US$ 1,000, child poverty rate in %* | | | | | | | | | | | |
|  | | | | | | | | | | | |

**Regression results**

| response interval (log) | Coef. | | St.Err. | t-value | | p-value | [95% Conf | | Interval] | | Sig |
| --- | --- | --- | --- | --- | --- | --- | --- | --- | --- | --- | --- |
| LL | 0 | | . | . | | . | . | | . | |  |
| Constant | 1.923 | | .045 | 42.47 | | 0 | 1.835 | | 2.012 | | *** |
|  | | | | | | | | | | | |
| Mean dependent var | | 1.927 | | | SD dependent var | | | 0.572 | |  |  |
| Overall r-squared | | 0.000 | | | Number of obs | | | 208 | |  |  |
| Chi-square | | . | | | Prob > chi2 | | | . | |  |  |
| R-squared within | | 0.000 | | | R-squared between | | | 0.000 | |  |  |
| **** p<.01, ** p<.05, * p<.1, MHI median household income in US$ 1,000, child poverty rate in %* | | | | | | | | | | | |
|  | | | | | | | | | | | |

**Regression results**

| on-scene interval | Coef. | | St.Err. | t-value | | p-value | [95% Conf | | Interval] | | Sig |
| --- | --- | --- | --- | --- | --- | --- | --- | --- | --- | --- | --- |
| LL | 0 | | . | . | | . | . | | . | |  |
| Constant | 13.918 | | .629 | 22.12 | | 0 | 12.685 | | 15.151 | | *** |
|  | | | | | | | | | | | |
| Mean dependent var | | 13.918 | | | SD dependent var | | | 8.625 | |  |  |
| Overall r-squared | | 0.000 | | | Number of obs | | | 188 | |  |  |
| Chi-square | | . | | | Prob > chi2 | | | . | |  |  |
| R-squared within | | 0.000 | | | R-squared between | | | 0.000 | |  |  |
| **** p<.01, ** p<.05, * p<.1, MHI median household income in US$ 1,000, child poverty rate in %* | | | | | | | | | | | |
|  | | | | | | | | | | | |

**Regression results**

| transport interval (log) | Coef. | | St.Err. | t-value | | p-value | [95% Conf | | Interval] | | Sig |
| --- | --- | --- | --- | --- | --- | --- | --- | --- | --- | --- | --- |
| LL | 0 | | . | . | | . | . | | . | |  |
| Constant | 2.202 | | .059 | 37.48 | | 0 | 2.087 | | 2.318 | | *** |
|  | | | | | | | | | | | |
| Mean dependent var | | 2.185 | | | SD dependent var | | | 0.673 | |  |  |
| Overall r-squared | | 0.000 | | | Number of obs | | | 181 | |  |  |
| Chi-square | | . | | | Prob > chi2 | | | . | |  |  |
| R-squared within | | 0.000 | | | R-squared between | | | 0.000 | |  |  |
| **** p<.01, ** p<.05, * p<.1, MHI median household income in US$ 1,000, child poverty rate in %* | | | | | | | | | | | |
|  | | | | | | | | | | | |

**Regression results**

| back-to-service interval (log) | Coef. | | St.Err. | t-value | | p-value | [95% Conf | | Interval] | | Sig |
| --- | --- | --- | --- | --- | --- | --- | --- | --- | --- | --- | --- |
| LL | 0 | | . | . | | . | . | | . | |  |
| Constant | 2.946 | | .104 | 28.36 | | 0 | 2.743 | | 3.15 | | *** |
|  | | | | | | | | | | | |
| Mean dependent var | | 2.985 | | | SD dependent var | | | 0.981 | |  |  |
| Overall r-squared | | 0.000 | | | Number of obs | | | 181 | |  |  |
| Chi-square | | . | | | Prob > chi2 | | | . | |  |  |
| R-squared within | | 0.000 | | | R-squared between | | | 0.000 | |  |  |
| **** p<.01, ** p<.05, * p<.1, MHI median household income in US$ 1,000, child poverty rate in %* | | | | | | | | | | | |
|  | | | | | | | | | | | |

**Regression results**

| overall mission interval | Coef. | | St.Err. | t-value | | p-value | [95% Conf | | Interval] | | Sig |
| --- | --- | --- | --- | --- | --- | --- | --- | --- | --- | --- | --- |
| LL | 0 | | . | . | | . | . | | . | |  |
| Constant | 55.026 | | 2.081 | 26.45 | | 0 | 50.948 | | 59.104 | | *** |
|  | | | | | | | | | | | |
| Mean dependent var | | 55.056 | | | SD dependent var | | | 24.339 | |  |  |
| Overall r-squared | | 0.000 | | | Number of obs | | | 208 | |  |  |
| Chi-square | | . | | | Prob > chi2 | | | . | |  |  |
| R-squared within | | 0.000 | | | R-squared between | | | 0.000 | |  |  |
| **** p<.01, ** p<.05, * p<.1, MHI median household income in US$ 1,000, child poverty rate in %* | | | | | | | | | | | |
|  | | | | | | | | | | | |
